# Comprehensive phenotypic analysis of the Dp1Tyb mouse strain reveals a broad range of Down syndrome-related phenotypes

**DOI:** 10.1242/dmm.049157

**Published:** 2021-10-18

**Authors:** Eva Lana-Elola, Heather Cater, Sheona Watson-Scales, Simon Greenaway, Jennifer Müller-Winkler, Dorota Gibbins, Mihaela Nemes, Amy Slender, Tertius Hough, Piia Keskivali-Bond, Cheryl L. Scudamore, Eleanor Herbert, Gareth T. Banks, Helene Mobbs, Tara Canonica, Justin Tosh, Suzanna Noy, Miriam Llorian, Patrick M. Nolan, Julian L. Griffin, Mark Good, Michelle Simon, Ann-Marie Mallon, Sara Wells, Elizabeth M. C. Fisher, Victor L. J. Tybulewicz

**Affiliations:** 1 The Francis Crick Institute, London NW1 1AT, UK; 2 MRC Harwell Institute, Harwell Campus, Didcot, OX11 0RD, UK; 3 Department of Biochemistry and Cambridge Systems Biology Centre, University of Cambridge, Cambridge CB2 1QW, UK; 4 School of Psychology, Cardiff University, Cardiff CF10 3AT, UK; 5 UCL Queen Square Institute of Neurology, London WC1N 3BG, UK; 6 Imperial College Dementia Research Institute, Imperial College London, London W12 7TA, UK; 7 Department of Immunology and Inflammation, Imperial College London, London W12 0NN, UK

**Keywords:** Down syndrome, Mouse model, Craniofacial development, Memory, Sleep, Hearing, Diabetes, Haematopoiesis

## Abstract

Down syndrome (DS), trisomy 21, results in many complex phenotypes including cognitive deficits, heart defects and craniofacial alterations. Phenotypes arise from an extra copy of human chromosome 21 (Hsa21) genes. However, these dosage-sensitive causative genes remain unknown. Animal models enable identification of genes and pathological mechanisms. The Dp1Tyb mouse model of DS has an extra copy of 63% of Hsa21-orthologous mouse genes. In order to establish whether this model recapitulates DS phenotypes, we comprehensively phenotyped Dp1Tyb mice using 28 tests of different physiological systems and found that 468 out of 1800 parameters were significantly altered. We show that Dp1Tyb mice have wide-ranging DS-like phenotypes, including aberrant erythropoiesis and megakaryopoiesis, reduced bone density, craniofacial changes, altered cardiac function, a pre-diabetic state, and deficits in memory, locomotion, hearing and sleep. Thus, Dp1Tyb mice are an excellent model for investigating complex DS phenotype-genotype relationships for this common disorder.

## Abstract (precis)

**Editor's choice:** Comprehensive phenotyping shows that Dp1Tyb mice, a model of Down syndrome (DS), have a broad range of DS-related phenotypes and can be used to investigate causative genes and their pathological mechanisms.

## INTRODUCTION

Down syndrome (DS), which arises from trisomy of human chromosome 21 (Hsa21), is a complex condition affecting many tissues with different severity and penetrance (Antonarakis et al., 2020). Individuals with DS have learning and memory impairment, motor deficits, disrupted sleep, shorter stature, reduced bone density, craniofacial alterations, congenital heart defects, leukaemia, diabetes, and impaired hearing and vision. People with DS are high risk for Alzheimer's disease (AD); ∼50% have signs of dementia by age 60 (Wiseman et al., 2015). With a prevalence of ∼1 in 800 live births, DS is the most common genetic cause of intellectual disability, AD and heart defects (Antonarakis, 2017; Wiseman et al., 2015).

Trisomy 21 was shown as the cause of DS in 1959 (Gautier and Harper, 2009; Jacobs et al., 1959; Lejeune et al., 1959), but there are still no effective treatments for most DS phenotypes, including cognitive aspects. Phenotypes likely result from increased dosage of Hsa21 genes, which comprise ∼230 coding genes, and many more non-coding elements (Antonarakis, 2017). Identification of dosage-sensitive Hsa21 genes causing DS phenotypes would facilitate studies of underlying pathological mechanisms, and development of new treatments. However, for most DS phenotypes, causative genes are unknown (Lana-Elola et al., 2011).

By modelling DS in mice, we can use the power of mouse genetics to find dosage-sensitive genes and so investigate pathology. Two approaches have been taken. First, mice have been generated with an extra human chromosome comprising most of Hsa21 (Tc1 and TcMac21 mice) (Kazuki et al., 2020; O'Doherty et al., 2005). Second, chromosome engineering has been used to create mouse strains with an additional copy of regions orthologous to Hsa21 (Herault et al., 2017; Moyer et al., 2020). The largest region of Hsa21 orthology (23 Mb) is on mouse chromosome (Mmu)16, with smaller regions on Mmu10 (3 Mb) and Mmu17 (2 Mb) (Herault et al., 2017). The Dp1Tyb and Dp1Yey strains have an extra copy of the entire Hsa21-orthologous region of Mmu16 (Lana-Elola et al., 2016; Li et al., 2007). Other strains have been generated with an extra copy of smaller Hsa21-orthologous regions, allowing mapping of causative genes (Brault et al., 2015; Duchon et al., 2008; Lana-Elola et al., 2016; Liu et al., 2014, 2011; Olson et al., 2004; Pereira et al., 2009; Raveau et al., 2012; Yu et al., 2010b).

However, although several DS mouse models have been investigated for individual phenotypes, there has been no comprehensive analysis to determine whether a single DS model recapitulates the breadth of DS phenotypes, validating them for the human condition.

The Dp1Tyb mouse strain has an extra copy of 148 coding genes on Mmu16, comprising 63% of Hsa21-orthologous genes in the mouse genome, and thus replicates the majority of the gene-dosage increase in DS, making it an excellent genetic model of the condition (Lana-Elola et al., 2016; Moyer et al., 2020). Here, we use the extensive tests in the International Mouse Phenotyping Consortium (IMPC) (Meehan et al., 2017), augmented with further bespoke assays, to comprehensively phenotype this model. We find that Dp1Tyb mice have significant changes in 468 out of 1800 parameters, displaying wide-ranging DS-like features, including decreased bone density, craniofacial changes, altered cardiac function, aberrant erythropoiesis and megakaryopoiesis, a pre-diabetic state, defective hearing, learning and memory deficits, impaired motor activity and disrupted sleep. Thus, Dp1Tyb mice faithfully recapitulate many of the complex phenotypes found in DS and are an excellent tool for discovering causative genes and their pathological mechanisms.

## RESULTS

### Phenotyping pipelines

**Fig. 1.**
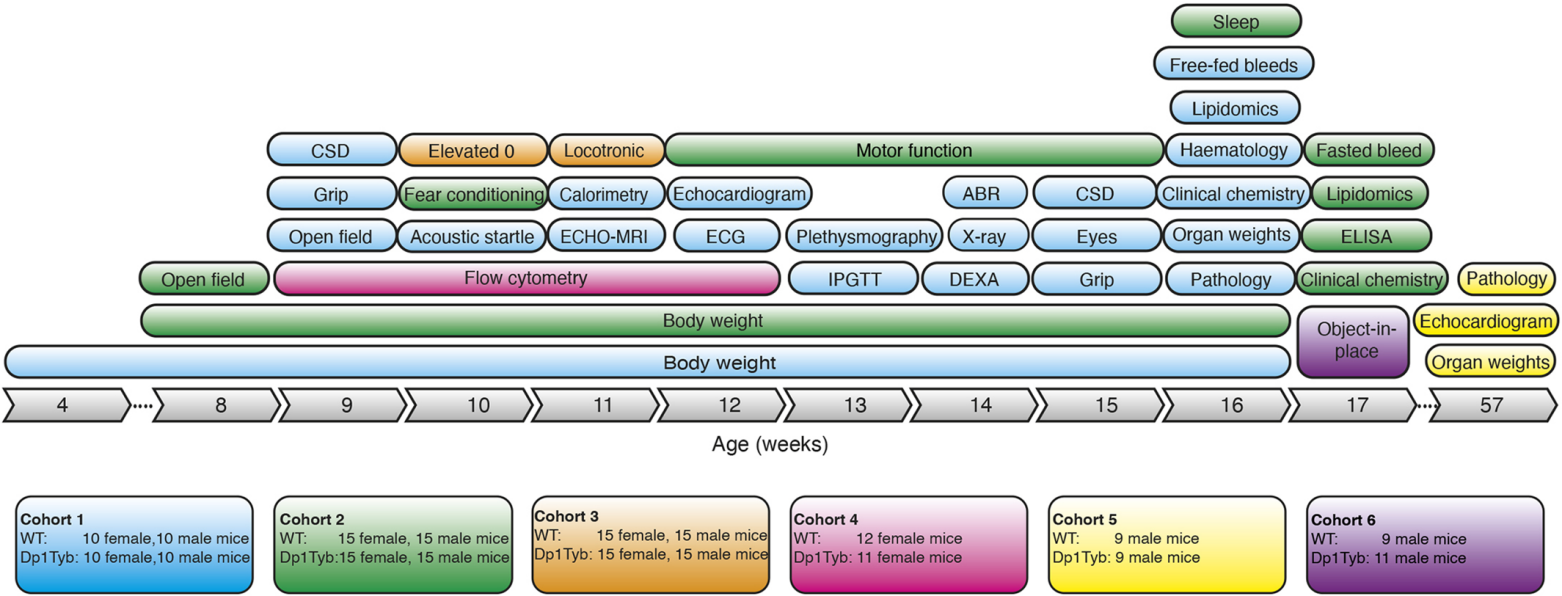
**Broad phenotyping pipeline used for analysis of Dp1Tyb mice.** Diagram shows the six cohorts of Dp1Tyb mice and wild-type (WT) controls that were used in the broad phenotyping analysis. Each cohort is indicated in a different colour, showing the tests it underwent, the numbers and sex of mice involved, and the age at which the tests were administered. ABR, acoustic brainstem response; CSD, Combined SHIRPA and Dysmorphology test; DEXA, dual energy X-ray absorption; ECG, electrocardiogram; Elevated 0, elevated 0 maze; ELISA, enzyme-linked immunosorbent assay for plasma hormones; IPGTT, intraperitoneal glucose tolerance test.

To carry out a broad phenotypic analysis, we generated Dp1Tyb and control wild-type (WT) animals on a C57BL/6J background, and analysed cohorts using wide-ranging procedures arranged into pipelines, with animals given specific tests at particular ages (Fig. 1). We used the IMPC pipeline (Meehan et al., 2017) to carry out a broad physiological analysis of Dp1Tyb and WT animals (cohort 1). Because cognitive and behavioural changes are DS hallmarks, we set up three pipelines for neurological aspects (cohorts 2, 3 and 6). We used flow cytometric analysis to investigate the haematopoietic system (cohort 4). Lastly, we analysed older mice, ∼1 year of age, for pathological changes and cardiac function (cohort 5).

In total, mice were analysed in 28 different procedures, generating data for 1800 parameters (where both sexes were used, males, females and both sexes together are counted as separate parameters). Statistical analysis showed that using a false discovery rate (FDR) of less than 5% (*q*≤0.05), Dp1Tyb mice had significant changes in 468 parameters from 22 procedures compared to WT mice (Table S1). Where there were differences between Dp1Tyb and WT mice, these were similar for both sexes, except for 15 parameters in six procedures showing significant sexual dimorphism (*q*≤0.05).

It has been suggested that aneuploidy results in greater phenotypic variability (Beach et al., 2017). We investigated the variance of the 1450 parameters that had a numerical value and found that just 39 parameters showed a significant difference in variance between the genotypes (*q*≤0.05), with 22 and 17 of these showing larger variation in WT and Dp1Tyb mice, respectively. There was no significant difference in the coefficient of variation for these 1450 parameters between either female or male Dp1Tyb mice compared to WT controls (Fig. S1A, Table S1). Thus, Dp1Tyb mice do not show greater phenotypic variation in the parameters measured in this study.

### Decreased viability of Dp1Tyb mice

Analysis at weaning from Dp1Tyb×C57BL/6J crosses showed that only 31% of the female pups and 38% of the male pups carried the Dp1Tyb mutation, significantly less than the expected 50% proportion (Fig. S1B). This implies that 54% of female Dp1Tyb pups and 39% of male Dp1Tyb pups had been lost prior to weaning. Because the proportion of Dp1Tyb embryos was not altered at embryonic day (E)14.5 of gestation (Lana-Elola et al., 2016), the loss must be occurring between E14.5 and weaning.

### Increased expression of duplicated genes in Dp1Tyb hippocampus

**Fig. 2.**
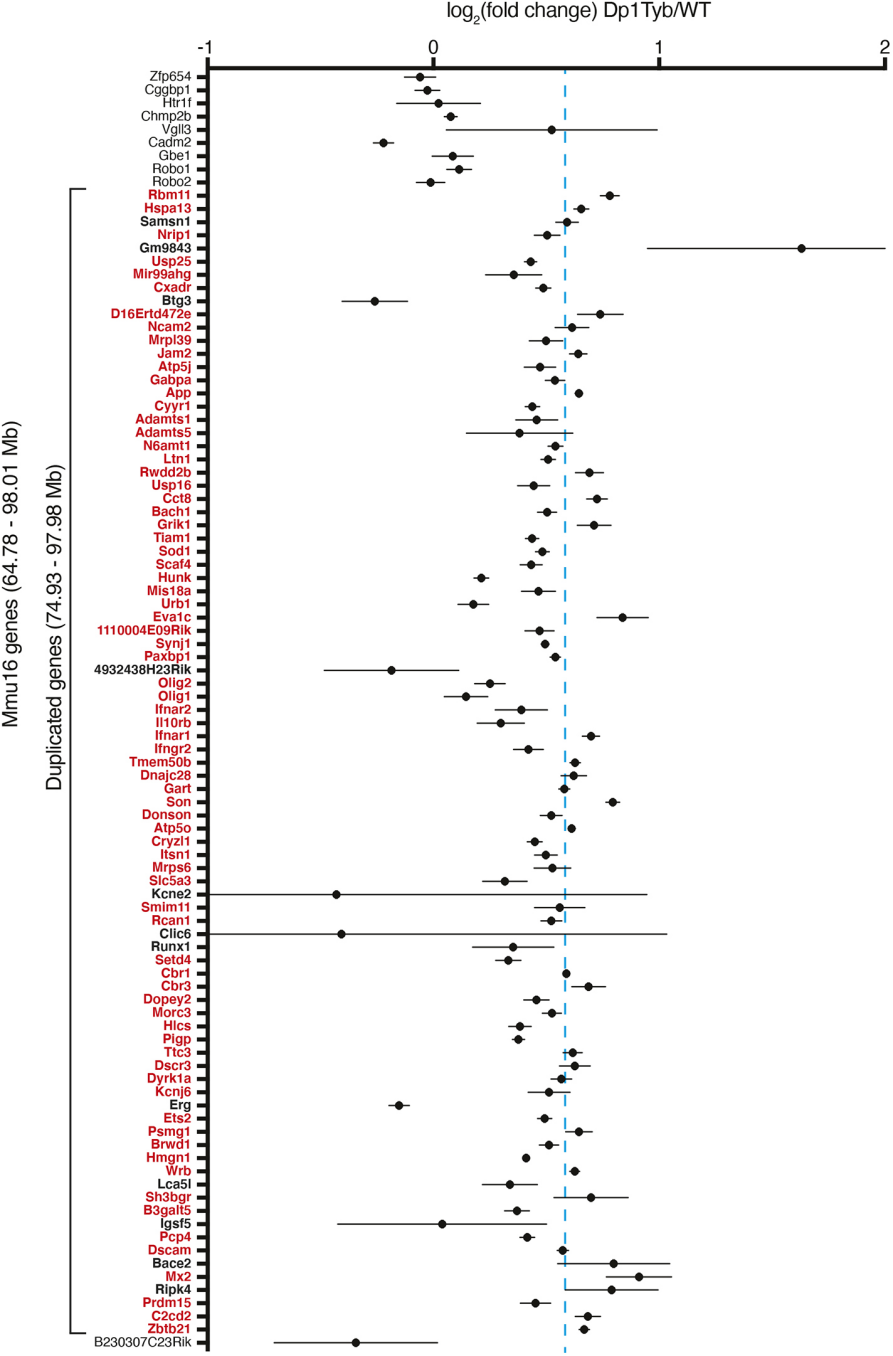
**Upregulation of expression of genes in duplicated region of Dp1Tyb mice.** Mean±s.e.m. log_2_ fold change in gene expression in the hippocampus between Dp1Tyb and WT mice (*n*=5 for each). Only expressed coding genes and one miRNA host gene shown; expressed genes were defined by the sum of expression over all ten samples >1 transcripts per million reads (TPM) and having a measured *P*-value in DEseq2. Genes within the duplicated region of Mmu16 are shown in bold. Genes showing a significantly different expression in Dp1Tyb compared to WT (adjusted *P*-value <0.05) are listed in red. Ten genes that are not duplicated (nine on the centromeric and one on the telomeric side of the duplication) are included in the figure to allow comparison with duplicated genes. The dashed blue line indicates a fold change of 1.5 expected by the increased dosage of the duplicated genes.

We investigated whether the additional 23 Mb of Mmu16 in Dp1Tyb mice results in increased expression of the 148 coding genes in this region. RNA sequencing of Dp1Tyb and WT adult hippocampus showed that, of the 87 expressed genes in the duplicated region, 75 were significantly upregulated and no genes were significantly downregulated (Fig. 2). The mean (±s.d.) upregulation was 1.43 (±0.28)-fold, similar to the expected 1.5-fold increase. Thus, the additional 23 Mb region of Mmu16 results in increased gene expression in line with gene copy number.

### Dp1Tyb mice have altered skeletal development

**Fig. 3.**
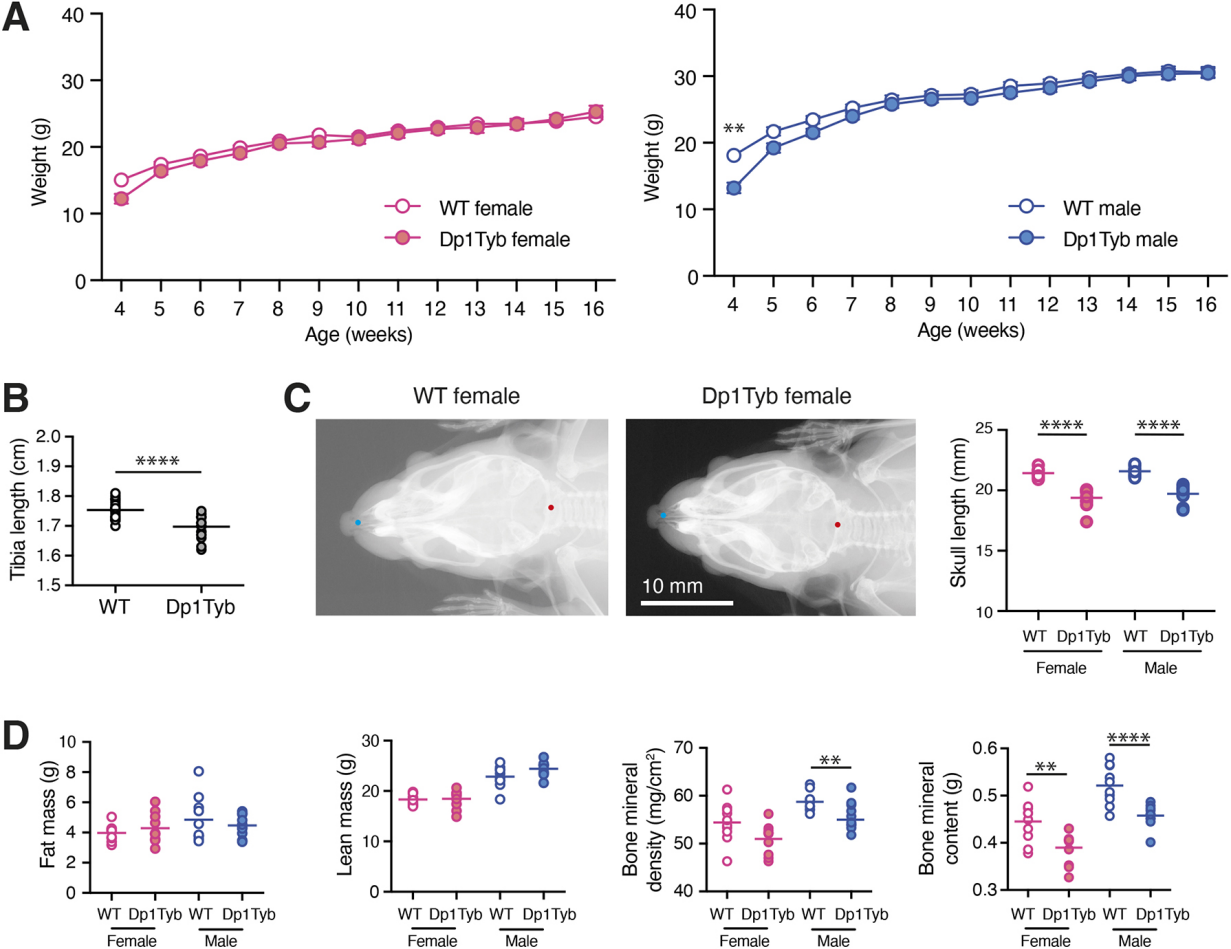
**Skeletal changes and decreased bone mineral density and content in Dp1Tyb mice.** (A) Mean±s.e.m. weight of Dp1Tyb and WT mice (cohort 1) as a function of age. (B) Tibia length of WT and Dp1Tyb mice (cohort 1), showing combined data from females and males, determined from X-ray images. (C) Example X-ray images of dorsal views of WT and Dp1Tyb female mouse skulls (cohort 1). Graph shows the length of the skull from the anterior tip of the nasal bone to the posterior of the occipital bone, which are indicated as blue and red dots on the images, respectively, from WT and Dp1Tyb mice (cohort 1). (D) Fat mass, lean mass, bone mineral density and bone mineral content of Dp1Tyb and WT mice (cohort 1) determined by DEXA. Horizontal lines indicate mean. Here and in other figures in which the sexes are analysed separately, the graphs are coloured pink and blue for females and males, respectively. Where both sexes are analysed together, graphs use white (WT) and grey (Dp1Tyb) to distinguish the genotypes. **0.001<*q*<0.01; ****q<0.0001.

Analysis of body weights from 4 to 16 weeks of age showed that male Dp1Tyb mice weighed less than WT mice at 4 weeks, but caught up afterwards, with no differences at later ages (Fig. 3A). There were no significant differences in the body weights of female Dp1Tyb and WT mice over the same timeframe.

People with DS are typically short in stature, have lower bone density and characteristic changes in craniofacial morphology, such as brachycephaly (front to back shortening of the skull), and increased body fat content (Baptista et al., 2005; Costa et al., 2018; Gutierrez-Hervas et al., 2020; Korenberg et al., 1994; LaCombe and Roper, 2020; Vicente et al., 2020). X-ray analysis at 14 weeks of age showed that Dp1Tyb mice had shorter tibia and skulls (Fig. 3B,C), and the Combined SHIRPA and Dysmorphology (CSD) analysis (Rogers et al., 1997) revealed that they had abnormally shaped heads, snouts and lips (Tables S2 and S3). Dual energy X-ray absorption (DEXA) showed that Dp1Tyb mice had reduced bone mineral density and content, without changes in fat or lean mass, which was confirmed by ECHO-MRI (Fig. 3D; Fig. S1C). Thus, Dp1Tyb mice have substantially altered bone growth resulting in skeletal dysmorphology.

### Dp1Tyb mice have enlarged spleens and no Aβ deposition in the hippocampus

Analysis of organ weights showed that Dp1Tyb mice at 16 weeks had slightly altered weights of heart and kidneys and at 57 weeks had reduced liver weights (Fig. S1D,E). However, the largest change was an increase in Dp1Tyb spleen weights at both ages (Fig. S1D,E). Histology showed increased extramedullary haematopoiesis in Dp1Tyb spleens (Fig. S2A, Table S4). Significant changes were also seen in the portal areas of the liver, with bile duct hyperplasia and vascular anomalies (Fig. S2B).

With the exception of ears (see ‘Dp1Tyb mice have otitis media and impaired hearing’ section), histological analysis of other organs in Dp1Tyb mice showed no significant findings, with any changes recorded being within the expected normal variation in pathology for the C57BL/6J background (Table S4). Given the high prevalence of AD in DS (Wiseman et al., 2015), we also examined the hippocampus of 1-year-old mice for the presence of amyloid-beta (Aβ). Whereas Aβ deposition was readily seen in the Aβ-overexpressing J20 mouse AD model, we detected no deposition in Dp1Tyb or control mice (Fig. S2C).

### Increased metabolic rate in Dp1Tyb mice

People with DS have a reduced resting metabolic rate (Allison et al., 1995; Luke et al., 1994). In contrast, indirect calorimetry showed that Dp1Tyb mice produced more CO_2_ (VCO_2_), used more O_2_ (VO_2_) and had higher heat production, indicating an increased metabolic rate and an elevated respiratory exchange ratio (RER) (Fig. S3A). The latter suggests that the mice may be using a higher ratio of carbohydrate to fat as a fuel source.

### Dp1Tyb mice have altered heart function

**Fig. 4.**
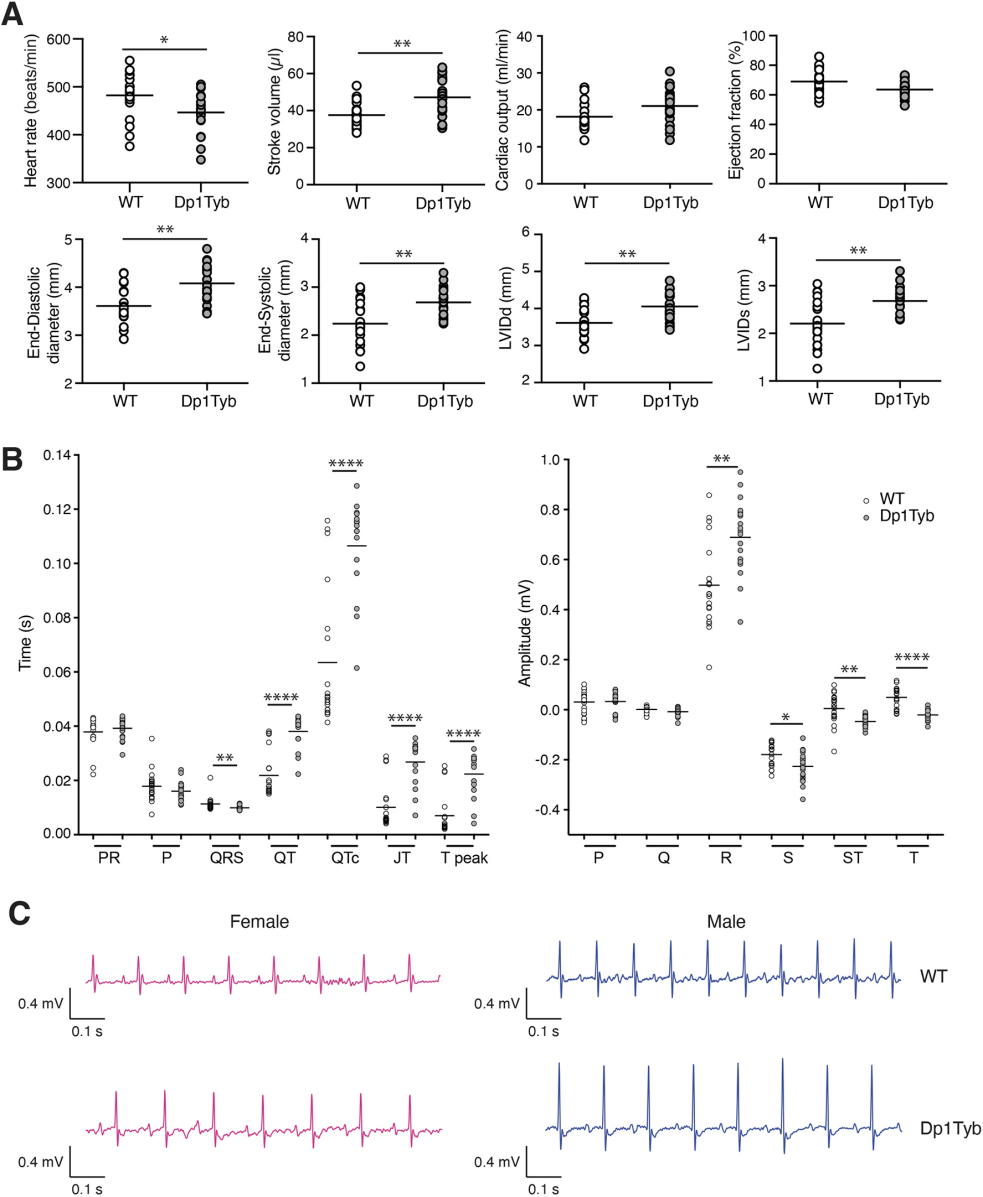
**Altered cardiac function in Dp1Tyb mice.** (A) Heart rate, stroke volume, cardiac output, ejection fraction, end-diastolic diameter, end-systolic diameter, left ventricular inner diameter in diastole (LVIDd) and left ventricular inner diameter in systole (LVIDs) in WT and Dp1Tyb mice (females and males combined) from cohort 1 determined by echocardiography. (B) Mean interval durations and wave amplitudes from electrocardiogram (ECG) analysis of WT and Dp1Tyb mice (females and males combined) from cohort 1. (C) Representative ECG traces. Horizontal lines indicate mean. *0.01<*q*<0.05; **0.001<*q*<0.01; ****q<0.0001.

Approximately 40% of neonates with DS have congenital heart defects, typically ventricular (VSD) or atrioventricular (AVSD) septal defects (Vis et al., 2009). Dp1Tyb embryos have a high prevalence of VSD and AVSD at E14.5 of gestation, resembling defects seen in DS (Lana-Elola et al., 2016). Analysis of heart function at 12 weeks by echocardiography showed that Dp1Tyb animals had a slower heart rate, increased stroke volume, increased cardiac output, increased end-diastolic and end-systolic diameters, and increased left ventricular internal diameter at diastole and systole compared to WT mice (Fig. 4A). Electrocardiogram (ECG) measurements showed longer QT, corrected QT (QTc), JT and T peak times, and larger amplitude of the R wave, consistent with increased left ventricle size (Fig. 4B,C). These changes were not progressive, because echocardiography showed no changes in cardiac function in 57-week-old Dp1Tyb mice (Fig. S3B).

### Increased breathing volumes in Dp1Tyb mice

**Fig. 5.**
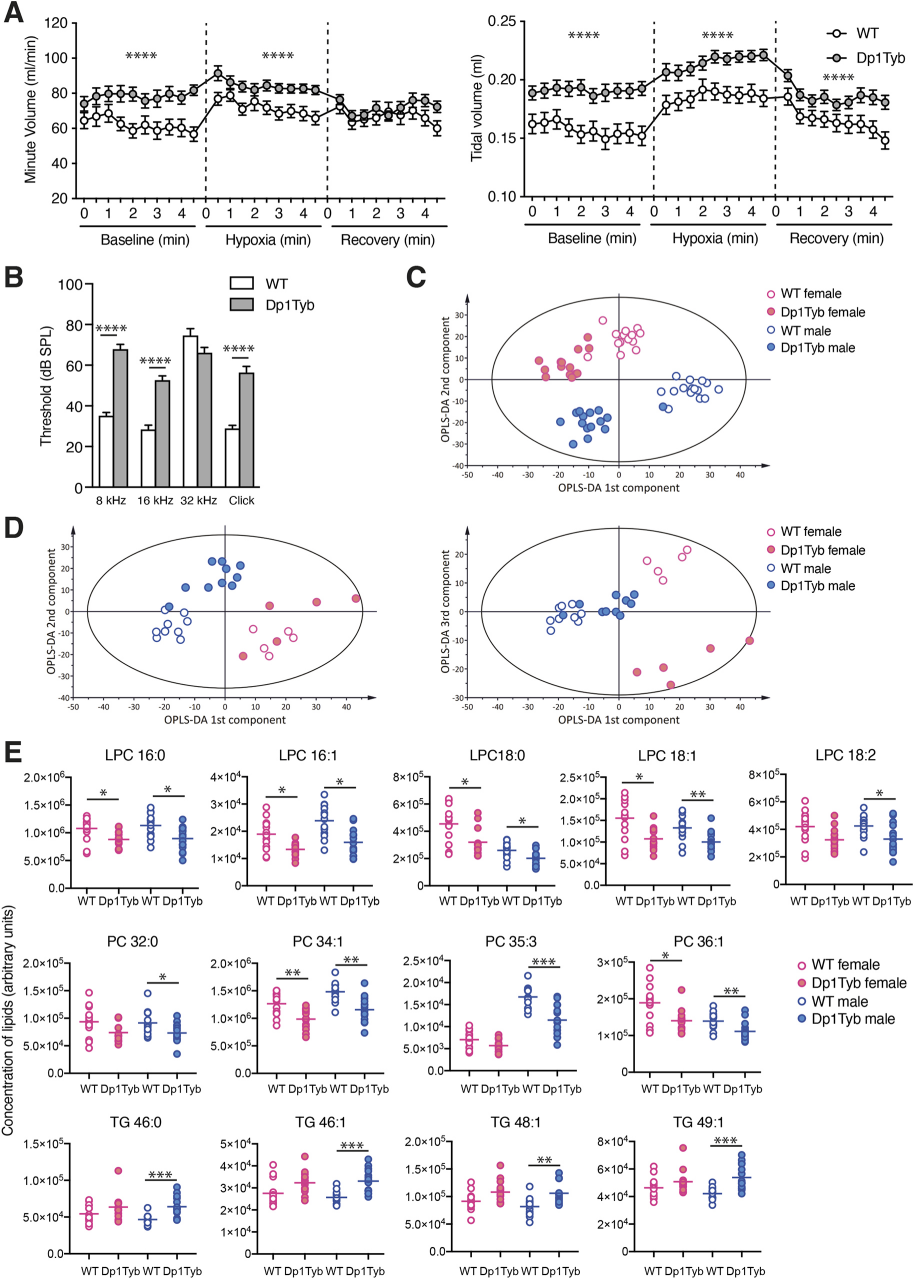
**Altered breathing, auditory brainstem response and plasma lipids in Dp1Tyb mice.** (A) Mean±s.e.m. volume of breaths over a minute or single breaths (tidal) taken by WT or Dp1Tyb mice (cohort 1, females and males combined) analysed by whole-body plethysmography over three periods of 5 min: baseline normoxia, hypoxia challenge (10% O_2_, 3% CO_2_) and recovery in normoxia. (B) Mean±s.e.m. auditory brainstem response of WT or Dp1Tyb mice from cohort 1 (females and males combined) at different frequencies (8, 16 and 32 kHz) and to a click box of mixed tones (Click). (C,D) Graphs of orthogonal partial least squares discriminant analysis (OPLS-DA) of lipids in plasma from fasted (cohort 2; C) and free-fed (cohort 1; D) mice, showing plots of 1st versus 2nd (C,D) or 1st versus 3rd (D) OPLS-DA components. The Hotelling T^2^ ellipse indicates the area within which 95% of the samples are expected to lie. (E) Plasma levels of the indicated lipids (arbitrary units) in fasted WT and Dp1Tyb mice (cohort 2). Horizontal lines indicate mean. *0.01<*q*<0.05; **0.001<*q*<0.01; ***0.0001<*q*<0.001; ****q<0.0001. LPC, lysophosphatidylcholine; PC, phosphatidylcholine; TG, triglyceride.

To analyse lung function, mice were placed into whole-body plethysmography chambers, and breathing was recorded for 5 min in normoxic conditions, then for 5 min in hypoxia (10% O_2_/3% CO_2_), before the mice were returned to normoxia for 5 min. WT and Dp1Tyb mice responded to hypoxia by increasing breathing volumes as measured per minute and per breath (tidal). However, under both normoxic and hypoxic conditions, Dp1Tyb mice had increased minute and tidal volumes compared to WT mice (Fig. 5A). This increased rate of breathing may be related to the increased O_2_ consumption.

### Dp1Tyb mice have otitis media and impaired hearing

More than half of children with DS have impaired hearing caused by otitis media, a middle-ear inflammation (Kreicher et al., 2018; Maris et al., 2014; Shott et al., 2001). Notably, pathological analysis showed that Dp1Tyb mice had otitis media (Table S4). To examine whether this might cause hearing deficits in Dp1Tyb mice, we analysed their auditory brainstem response and found that, compared to WT controls, Dp1Tyb mice had substantially higher minimum sound intensity thresholds required to elicit a brainstem response when challenged with sounds at 8 kHz and 16 kHz and with clicks consisting of mixed frequencies (Fig. 5B), indicating impaired hearing.

Children and adults with DS have an increased prevalence of eyesight defects in multiple eye structures, including the lid, iris, cornea, lens and retina (Krinsky-McHale et al., 2014). We examined the eyes of Dp1Tyb mice but found no visible defects within the eye (Table S5).

### Dp1Tyb mice show characteristics of a pre-diabetic state

DS is associated with increased prevalence of type 1 and type 2 diabetes (Alexander et al., 2016; Anwar et al., 1998; Bergholdt et al., 2006; Johnson et al., 2019). Thus, we measured the ability of Dp1Tyb and WT mice to clear an intraperitoneal injection of glucose and found no difference; thus, Dp1Tyb mice are not diabetic at 13 weeks of age (Fig. S3C).

We collected plasma from free-fed and fasted mice, and measured levels of multiple analytes and hormones. We found reduced levels of glucose in fasted, but not free-fed, Dp1Tyb male mice (Fig. S3D,E). Other analytes were unaffected, except for raised levels of inorganic phosphorus and aspartate aminotransferase (AST), and decreased alpha-amylase in free-fed mice, and increased glucagon and adiponectin in fasted mice (Fig. S3D,F). A slightly elevated AST/alanine aminotransferase (ALT) ratio (1.66 in Dp1Tyb mice versus 1.19 in WT mice; Table S1) may indicate liver pathology (Salaspuro, 1987), but this will require further investigation. Increased adiponectin has been reported in people with DS (Corsi et al., 2009).

We used mass spectrometry to measure levels of lipids in plasma from free-fed and fasted mice. Orthogonal partial least squares discriminant analysis (OPLS-DA) showed that, for both free-fed and fasted mice, lipid composition was distinct between Dp1Tyb and WT mice and between males and females (Fig. 5C,D). The differences in lipid composition were larger for fasted mice, with 85 out of 285 lipids showing a significant difference, compared to 11 out of 285 lipids in free-fed mice (Table S1). Dp1Tyb mice had significantly reduced levels of many species of both saturated and unsaturated lysophosphatidylcholine (LPC) and phosphatidylcholine (PC) lipids, and increased levels of many triglycerides (Fig. 5E). Decreased levels of LPC phospholipids and increased triglycerides are associated with a pre-diabetic state (Barber et al., 2012; Ferrannini et al., 2013; Kotronen et al., 2009; Suvitaival et al., 2018; Wang-Sattler et al., 2012). Furthermore, higher plasma triglycerides are found in people with non-alcoholic fatty liver disease (NAFLD) and in hepatic steatosis, a clinical subtype of NAFLD (Orešič et al., 2013; Sanders et al., 2018), suggesting that Dp1Tyb mice have characteristics of a pre-diabetic state with associated liver pathology.

### Increased erythropoiesis and megakaryopoiesis in Dp1Tyb mice

Ten to fifteen percent of neonates with DS present with transient abnormal myelopoiesis (TAM), a pre-leukaemic condition characterised by an accumulation of circulating megakaryoblasts (Bhatnagar et al., 2016; Garnett et al., 2020). Most children with TAM undergo spontaneous regression, but, in 10-20% of cases, the condition progresses to an acute megakaryoblastic leukaemia (Bhatnagar et al., 2016; Garnett et al., 2020). In addition, children with DS have a 20-fold higher risk of developing acute lymphoblastic leukaemia (DS-ALL), usually derived from B-cell progenitors (Lee et al., 2016).

**Fig. 6.**
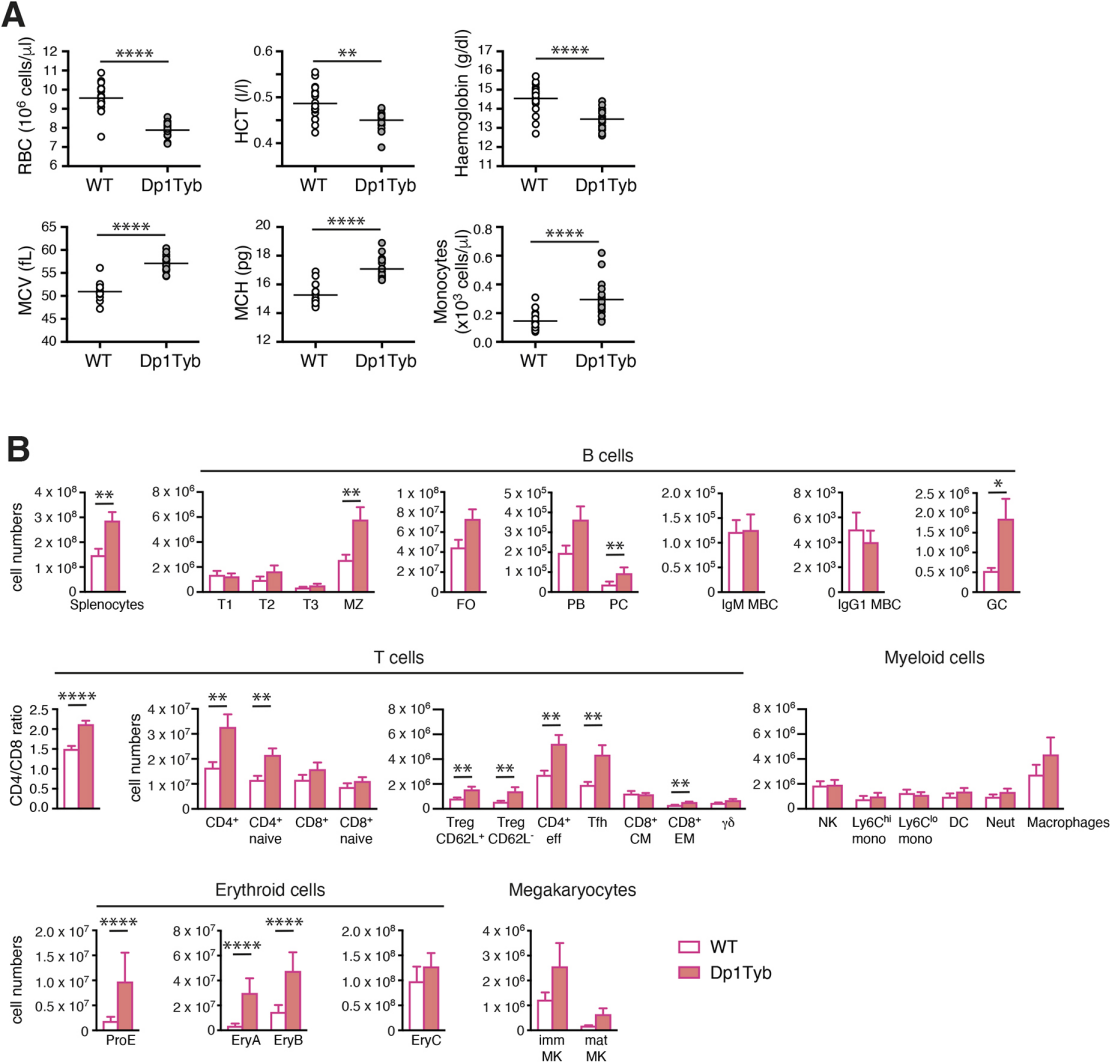
**Macrocytic anaemia, splenomegaly, and increased erythropoiesis and megakaryopoiesis in Dp1Tyb mice.** (A) Red blood cell (RBC) concentrations, haematocrit (HCT), haemoglobin, mean corpuscular volume (MCV), mean corpuscular haemoglobin (MCH) and monocytes in the blood of WT or Dp1Tyb mice (cohort 1, females and males combined). (B) Flow cytometric analysis of splenocytes from female WT and Dp1Tyb mice (cohort 4), showing mean±s.e.m. numbers of splenocytes, transitional type (T)1, T2, T3, marginal zone (MZ), follicular (FO), plasmablasts (PB), plasma cells (PC), IgM and IgG1 memory B cells (MBC), and germinal centre (GC) B cells; mean±s.e.m. ratio of CD4^+^/CD8^+^ T cells; and mean±s.e.m. numbers of total or naive CD4^+^ or CD8^+^ T cells, CD62L^+^ or CD62L^−^ regulatory T cells (Treg), effector T cells, T follicular helper (Tfh) cells, CD8^+^ central memory (CM) and effector memory (EM) T cells, γδ T cells, natural killer (NK) cells, Ly6C^hi^ and Ly6C^lo^ monocytes (mono), dendritic cells (DC), neutrophils (Neut), macrophages, pro-erythroblasts (ProE), EryA, EryB and EryC erythroid progenitors, and immature (imm) and mature (mat) megakaryoblasts (MK). *0.01<*q*<0.05; **0.001<*q*<0.01; ****q<0.0001.

Haematological analysis showed that Dp1Tyb mice have a macrocytic anaemia characterised by reduced numbers of erythrocytes, a reduced haematocrit and reduced haemoglobin concentration, but larger erythrocytes with more haemoglobin per cell (Fig. 6A). Analysis of other haematological parameters showed no changes, except for an increase in monocytes (Fig. 6A; Fig. S4).

We carried out comprehensive flow cytometric analysis of haematopoietic cells in the bone marrow, thymus, spleen, lymph nodes, peritoneal cavity and blood of Dp1Tyb and control mice (Figs S5 and S6). Dp1Tyb bone marrow had unchanged numbers of erythroid progenitors and immature and mature megakaryocytes (Fig. S7A), but reduced percentages of developing B-lineage subsets (pro-B, pre-B, immature and mature B cells) (Fig. S7B). Dp1Tyb mice had an increased number of splenocytes (Fig. 6B), in keeping with the increased splenic size (Fig. S1D). Many splenic lymphoid subsets were altered, including increased numbers of marginal zone B cells, germinal centre B cells, plasma cells, and multiple subsets of CD4^+^ T cells – naïve, effector, regulatory and T follicular helper T cells (Fig. 6B). However, the largest changes were substantial increases in the numbers and percentages of splenic pro-erythroblasts (ProE), EryA and EryB erythroid progenitors, and immature and mature megakaryocytes (Fig. 6B; Fig. S8), explaining the increased extramedullary haematopoiesis of Dp1Tyb spleens (Fig. S2A, Table S4).

Blood analysis showed no changes in percentages of leukocytes, including monocytes (Fig. S9A), whereas in Dp1Tyb peripheral and mesenteric lymph nodes we found small increases in B cells, and small reductions in CD8^+^ and γδ T cells (Fig. S9B,C). In the peritoneal cavity, conventional B2 cells were increased, with reduced percentages of B1a cells (Fig. S9D). Finally, analysis of developing T cell subsets in the thymus showed that Dp1Tyb mice had reduced numbers and percentages of the early double negative (DN)1, DN2 and DN4 cells, but increased numbers of intermediate and mature CD8^+^ single positive thymocytes (Fig. S10A,B). Thus, Dp1Tyb mice have increased erythropoiesis and megakaryopoiesis in the spleen and increases in some B and T cell subsets. Other lymphoid tissues showed small changes in B and T cell subsets.

### Dp1Tyb mice have impaired short-term associative memory

DS is the most common genetic cause of intellectual disability (Grieco et al., 2015), arising from cognitive impairment, with delayed acquisition of many developmental milestones (Lott and Dierssen, 2010). Children and adults with DS have a decreased ability to learn and show deficits in both short-term and long-term memory. DS results in poorer processing of verbal information, slower language acquisition, impaired attention, poor response inhibition and slower task planning.

**Fig. 7.**
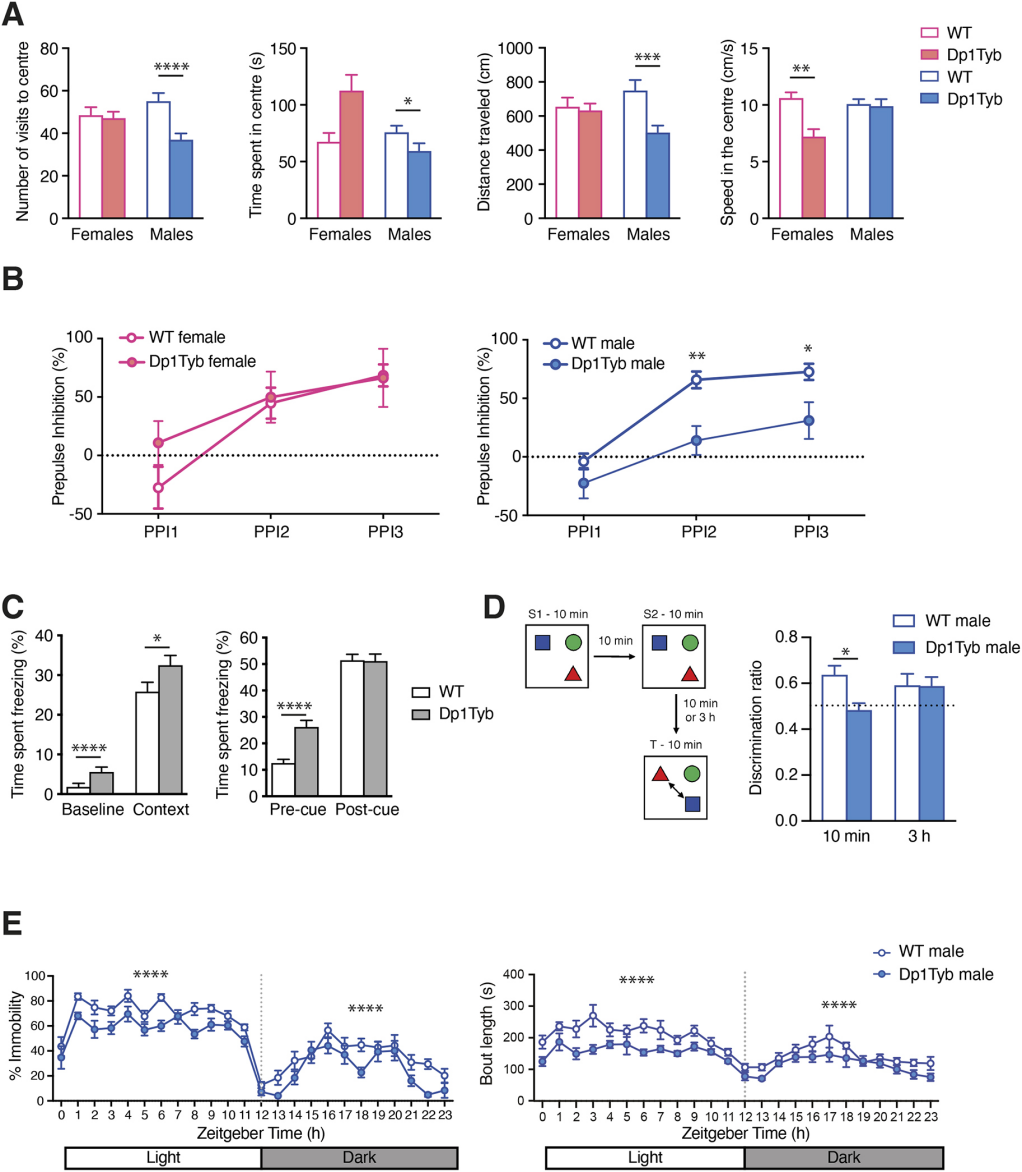
**Decreased open field activity, sleep and object-in-place memory in Dp1Tyb mice.** (A) Mean±s.e.m. number of visits in the centre, time spent in the centre, distance travelled in the centre and speed in the centre of mice in cohort 2 undergoing the open field test. (B) Mean±s.e.m. pre-pulse inhibition in WT and Dp1Tyb mice (cohort 1) of a response to a startle tone (110 dB) to increasing volumes of pre-pulse tones (55, 65, 70 dB; PPI1 to PPI3). (C) Fear conditioning test on WT and Dp1Tyb mice in cohort 2, showing mean±s.e.m. time spent freezing in response to being placed in a chamber associated with aversive experience (context) compared to response to the same chamber before conditioning (baseline), and also freezing time in a novel chamber before (pre-cue) or after (post-cue) being given an auditory tone associated with an aversive experience. (D) WT and Dp1Tyb mice (cohort 6) were placed in an arena with three different objects for two 10 min sampling periods (S1, S2), then tested after a delay of either 10 min or 3 h for their ability to recognise which objects had changed location (T, test phase). There were no differences between groups in object contact times during the sample phase. The discrimination ratio (mean±s.e.m.) measures the preferential interaction with switched objects compared to the unchanged object for mice tested after a 10 min or 3 h delay. A ratio of 0.5 (dotted line) indicates performance at chance level, with no associative object-in-place memory. (E) Mean±s.e.m. percentage immobility or length of immobile bouts of WT and Dp1Tyb mice (cohort 2) as a function of time, showing the light and dark phases of a 24 h period. Immobility is taken as a proxy of sleep. *0.01<*q*<0.05; **0.001<*q*<0.01; ***0.0001<*q*<0.001; ****q<0.0001.

We analysed Dp1Tyb mice in behavioural tests. The open field test assesses anxiety, activity and exploratory behaviour: mice are placed in an arena and monitored for the time they spend in the centre of the arena compared to the periphery, as well as movement speed and distance travelled. Compared to controls, Dp1Tyb mice visited the centre of the arena less frequently, spent less time in the centre, moved at slower speeds and travelled shorter distances (Fig. 7A). Thus, Dp1Tyb mice are less active, and the reduced time spent in the centre indicates that they may be more anxious, although this could also be the result of reduced movement. This analysis was extended using an elevated zero maze, which assesses the conflict between exploratory behaviour of novel environments and avoidance of well-lit open areas. This showed no difference between Dp1Tyb and WT mice in the time spent in open versus closed arms of the maze, implying that, at least in this assay, Dp1Tyb mice do not have increased anxiety (Fig. S11A). This apparent difference in anxiety may be due to differences in anxiolytic drivers between the two tests.

Dp1Tyb and control mice were assessed using the pre-pulse inhibition (PPI) of acoustic startle test, which measures sensorimotor gating mechanisms through exposing mice to a loud startle sound, which may be preceded by a quieter pre-pulse tone. As the intensity of the pre-pulse tone increases, the neural response to the following startle noise is hindered, resulting in decreased flinching. Such PPI is often impaired in neurological conditions such as schizophrenia (Braff et al., 2001). Dp1Tyb and WT mice showed increased PPI with increasing pre-pulse tone intensity, but male Dp1Tyb mice were significantly impaired in this response (Fig. 7B). Because Dp1Tyb mice have impaired hearing (Fig. 5B), it is unclear whether the decreased PPI is due to this sensory deficit or to defective sensorimotor gating.

In a fear conditioning test, we assessed memory of an aversive foot shock experience, determining whether the animal can associate it with context, a novel chamber, or with a cue in the form of an auditory conditioned stimulus. Responses to the context or cue were measured by the time the animal spent freezing. Dp1Tyb mice responded at least as well as WT mice to both context and cue, indicating no defect in associative memory of the aversive response (Fig. 7C). However, Dp1Tyb mice spent more time freezing when placed into novel chambers (baseline and pre-cue responses), again suggesting that they may be more anxious.

We measured spatial memory using an object-in-place test. The mice were placed in an arena with three distinct objects for two 10 min sample phases, 10 min apart. The mice were then removed for either 10 min or 3 h before being placed back in the arena for the test phase, in which the locations of two of the objects had been switched. Contact times with objects that had been moved were compared to contact time with the object that had not been moved using a discrimination ratio. We found that Dp1Tyb mice performed worse than WT mice when placed back into the arena after a 10 min delay – unlike WT mice, their discrimination ratio was not significantly different from chance (0.5) (Fig. 7D). Conversely, Dp1Tyb mice performed as well as WT mice after a 3 h delay. Thus, in this paradigm, Dp1Tyb mice have impaired short-term (10 min) but not longer-term (3 h) associative recognition memory.

### Dp1Tyb mice have disrupted sleep

A notable co-morbidity in DS is sleep disruption, characterised by poor sleep initiation and maintenance, both of which may contribute to cognitive impairment (Grieco et al., 2015). We assessed sleep in Dp1Tyb mice by tracking movement over a 24 h period in a 12 h light/12 h dark cycle, scoring cumulative periods of immobility of >40 s, which show high correlation with sleep (Fisher et al., 2012). Compared to WT mice, this cumulative immobility score was substantially reduced in Dp1Tyb mice, while immobility bout lengths were significantly shorter, particularly during the light phase, in which most sleep is scored (Fig. 7E). Thus, Dp1Tyb mice sleep less than WT controls and show a higher number of short-duration bouts, indicating a disrupted sleep pattern.

### Dp1Tyb mice have impaired motor function

**Fig. 8.**
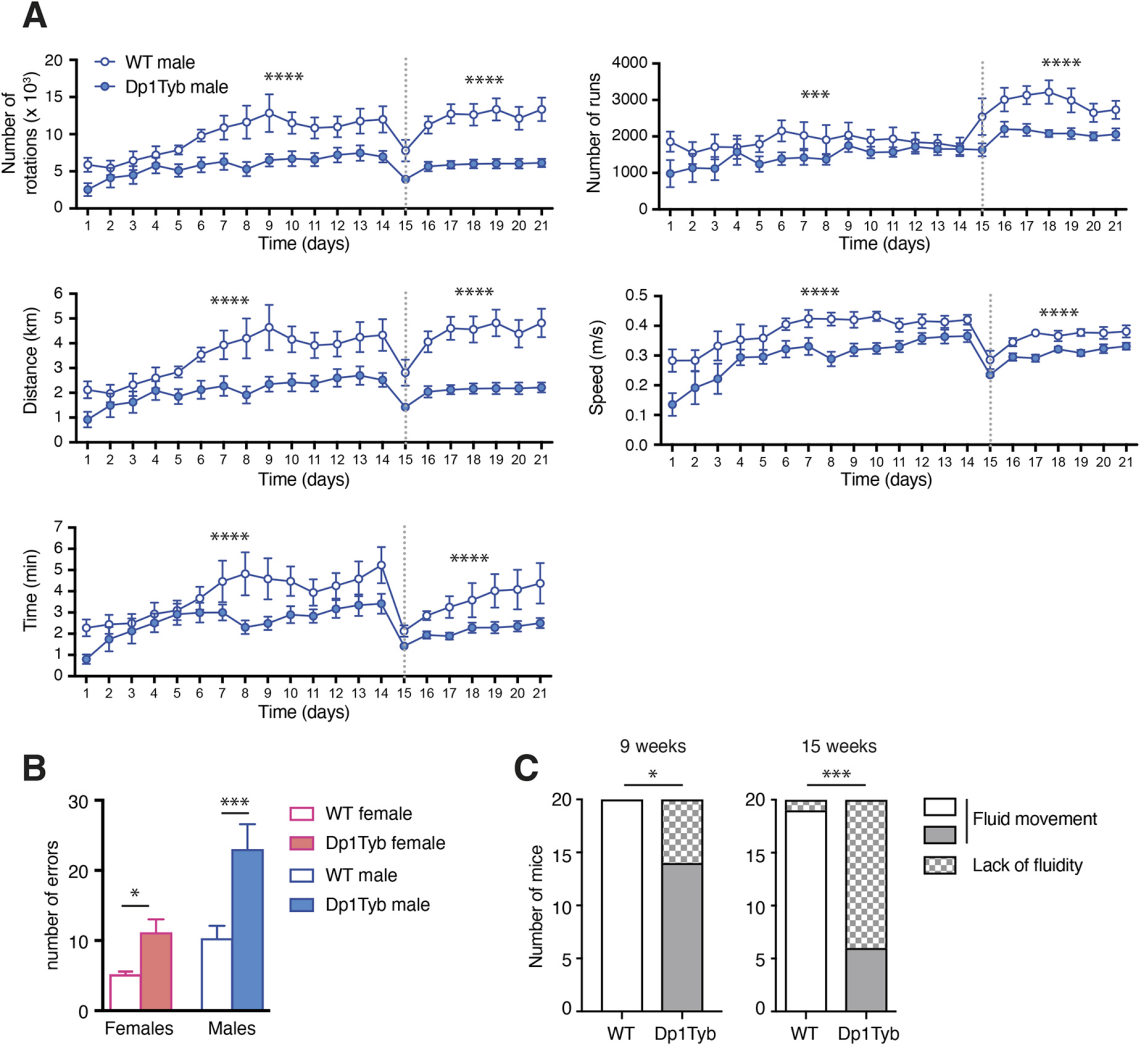
**Impaired locomotor activity in Dp1Tyb mice.** (A) Analysis of home cage wheel running by WT and Dp1Tyb mice (cohort 2) over a 3-week period, showing mean±s.e.m. number of rotations, number of runs, distance run, speed and time spent running. Dotted line indicates transition from a simple to a complex wheel in which several rungs were missing. (B) Mean±s.e.m. number of foot errors made by WT and Dp1Tyb mice (cohort 3) traversing a horizontal ladder in a Locotronic apparatus. (C) Graph of numbers of WT and Dp1Tyb mice (cohort 1, females and males combined) showing or lacking fluid movement as determined a CSD test at 9 and 15 weeks of age. *0.01<*q*<0.05; ***0.0001<*q*<0.001; ****q<0.0001.

Children with DS have impaired motor skills and postural control (Cardoso et al., 2015; Malak et al., 2015; Spanò et al., 1999). We assessed motor function in Dp1Tyb mice using multiple assays. Dp1Tyb mice spent less time in spontaneous wheel running, carrying out fewer runs and rotations, running at lower speeds and for shorter distances (Fig. 8A). In the Locotronic test, in which mice walk along a horizontal ladder with evenly spaced rungs, Dp1Tyb mice made more errors, indicating defects in motor coordination (Fig. 8B). Defective movement of Dp1Tyb mice was also seen in the CSD test (Fig. 8C). Dp1Tyb mice had normal grip strength, indicating that altered locomotor activity was not due to altered muscle tone (Fig. S11B). Thus, Dp1Tyb mice have substantially impaired motor function.

## DISCUSSION

To understand pathological mechanisms in DS, we need animal models that reflect the genetic and phenotypic complexity of this chromosomal disorder. Comprehensive phenotyping of the Dp1Tyb mouse model of DS shows significant differences compared to WT mice in 468 out of 1800 parameters across 22 different tests. Thus, an extra copy of 148 genes has substantial phenotypic consequences in many physiological domains. Importantly, many of the phenotypes in Dp1Tyb mice resemble those in people with DS (Table S6).

The reduced perinatal viability of Dp1Tyb pups may be caused by congenital heart defects. At E14.5, ∼25% of Dp1Tyb embryos have AVSD and a further 35% have VSD (Lana-Elola et al., 2016), similar to the defects reported in ∼40% of babies with DS. It is possible that pups with AVSD die perinatally, potentially accounting for the reduced viability.

Analysis of skeletal structures showed that Dp1Tyb mice have reduced bone density, shorter tibia and altered craniofacial structures, in particular brachycephaly, all of which are typical of DS (Baptista et al., 2005; Costa et al., 2018; Herrera et al., 2020; LaCombe and Roper, 2020; Vicente et al., 2020). In agreement with this, Dp1Tyb mice have shorter body and femur length (Thomas et al., 2020), smaller crania and mandibles, mid-facial hypoplasia and cranial doming (Toussaint et al., 2021). Similar craniofacial changes have been seen in the Dp1Yey mouse strain (Starbuck et al., 2014; Takahashi et al., 2020), which, like Dp1Tyb mice, was generated by Cre/loxP chromosome engineering and has an extra copy of the same Hsa21-orthologous genes as Dp1Tyb (Li et al., 2007). Indeed, where comparable analysis has been carried out, Dp1Tyb and Dp1Yey mice have very similar phenotypes (Table S6).

ECG studies in DS individuals without congenital heart defects showed increased T-wave length, similar to that seen in Dp1Tyb mice (Küçük et al., 2018). Cardiac function was altered in 12-week-old Dp1Tyb mice, which had a slower heart rate, and increased cardiac output, stroke volume and left ventricular diameters compared to WT mice. Increased cardiac output and stroke volume could be caused by abnormal fluid loading from a left-right shunt such as a septal defect. However, this would be expected to cause an increased heart rate. A more likely explanation is that a shunt during embryonic development would reduce the effective stroke volume, leading to renal hypoperfusion and compensatory salt and water retention to increase circulatory volume. If the shunt closed perinatally, the increased circulatory volume would result in the observed increased stroke volume and cardiac output with a reactive lower heart rate. Subsequent diuresis may have normalised the circulatory volume, thereby resulting in normal cardiac function in the older 57-week-old mice.

Lipidomic and clinical chemistry analysis of plasma showed that Dp1Tyb mice have characteristics of a pre-diabetic state with hepatic steatosis, conditions that are seen in people with DS (Dierssen et al., 2020). Further work is needed to determine whether the animals could be induced to develop glucose intolerance, for example by being fed a high-fat diet.

Dp1Tyb mice have a macrocytic anaemia alongside increased splenic erythropoiesis and megakaryopoiesis, resembling a pre-leukaemic condition. However, they do not develop overt megakaryoblastic leukaemia, likely because progression to leukaemia requires mutations in *Gata1* and other genes (Bhatnagar et al., 2016; Garnett et al., 2020). Similar macrocytic anaemia and elevated megakaryopoiesis are found in several other mouse DS models carrying three copies of Hsa21-orthologous Mmu16 genes (Alford et al., 2010; Carmichael et al., 2009; Kirsammer et al., 2008). We detected no DS-ALL; however, this leukaemia also requires additional mutations (Lee et al., 2016).

Sensory deficits, including in hearing and vision, are often seen in DS (Grieco et al., 2015). Dp1Tyb mice have defective hearing, which is likely due to otitis media, a common condition in DS (Kreicher et al., 2018; Maris et al., 2014; Shott et al., 2001). We found no eye defects in the mice, but further studies are needed to evaluate visual acuity. In a previous study, we found no defects in nociception or proprioception in Dp1Tyb mice (Watson-Scales et al., 2018).

Dp1Tyb mice have defective short-term memory, disrupted sleep and motor deficits, neurological features that have also been reported in people with DS (Cardoso et al., 2015; Grieco et al., 2015; Malak et al., 2015; Spanò et al., 1999). Previously we showed that these animals have reduced theta-wave frequency in the medial pre-frontal cortex and the hippocampus, and increased theta to high-gamma phase-amplitude coupling in the hippocampus, along with deficits in spatial working memory (Chang et al., 2020). Memory and motor deficits have also been reported in the related Dp1Yey mice (Aziz et al., 2018; Pinto et al., 2020; Watson-Scales et al., 2018; Yu et al., 2010a). The motor deficits in these mice may be partly due to motor neuron loss, which we also found in human DS (Watson-Scales et al., 2018). The disrupted sleep of Dp1Tyb mice may have a neurological basis, but analysis of Dp1Yey mice showed reduced upper-airway volume, suggesting that apnoea may affect sleep in these animals (Takahashi et al., 2020).

AD in DS is most likely caused by a third copy of the *APP* gene on Hsa21 (Wiseman et al., 2015). Dp1Tyb mice have three copies of *App* but show no deposition of Aβ in the hippocampus. This is not surprising, because amyloid pathology is only seen in mice expressing mutant amyloidogenic human APP proteins; overexpression of wild-type mouse APP is not sufficient (Myers and McGonigle, 2019).

Muscle hypotonia and increased body fat are features of DS (Antonarakis et al., 2004; Gutierrez-Hervas et al., 2020), but neither was seen in the Dp1Tyb mice. Furthermore, Dp1Tyb mice have higher VO_2_, VCO_2_, breathing rates and RER, in contrast to people with DS, who show lower VO_2_ and VCO_2_, and reduced breathing rates and RER (Boonman et al., 2019). Thus, Dp1Tyb mice do not model the reduced cardiorespiratory function in DS. These ‘missing’ phenotypes may be due to increased dosage of Hsa21 genes for which orthologues are in the Mmu10 and Mmu17 regions. To investigate this, a broad phenotypic analysis of mice that have extra copies of these regions will be needed (Yu et al., 2010b). Furthermore, the analysis presented here was carried out on a single genetic background (C57BL/6J). It is possible that analysis on other backgrounds would reveal further phenotypes.

In summary, we have performed the first comprehensive phenotypic analysis of a single mouse model for DS, revealing that Dp1Tyb mice have many DS-like phenotypes and thus can be used to investigate complex underlying pathogenetic mechanisms. Importantly, the existence of a complete panel of mouse strains with extra copies of shorter regions of Mmu16 will allow mapping and identification of the causative dosage-sensitive genes (Lana-Elola et al., 2016).

## MATERIALS AND METHODS

### Mice

C57BL/6J.129P2-Dp(16Lipi-Zbtb21)1TybEmcf (Dp1Tyb) mice (Lana-Elola et al., 2016) were bred in specific-pathogen free conditions at the Mary Lyon Centre (MRC Harwell) and at the Francis Crick Institute by backcrossing to C57BL/6J mice. All mice analysed had been backcrossed to the C57BL/6J background for at least ten generations. Mice in cohorts 1 and 2 were weighed once a week for the duration of each phenotyping pipeline. All tests were carried out by experimenters who were blind to the genotype of the mouse. *Zbtb20*^Tg(PDGFB-APPSwInd)20Lms^ mice (J20) (Mucke et al., 2000) were bred on a C57BL/6J background at the UCL Institute of Neurology. All animal work was carried out under Project Licences granted by the UK Home Office, thereby complying with relevant national regulations.

### Analysis of RNA-sequencing data

Using our RNA-sequencing data from the hippocampi of five WT and five Dp1Tyb mice aged 18.5-19 weeks (Ahlfors et al., 2019), we calculated the fold change in expression for the genes in the duplicated region on Mmu16 (*Lipi* to *Zbtb21*), using the transcripts per million reads (TPM) measure, excluding genes for which expression summed over all ten samples was <1 TPM. Significantly differentially expressed genes were identified using DEseq2 (Love et al., 2014).

### IMPC pipelines

The pipeline of tests for cohort 1 is closely based on those used by the IMPC pipeline (Meehan et al., 2017). More detailed descriptions of these protocols are available on the IMPRESS website (www.mousephenotype.org/impress).

### X-ray analysis

X-ray images of the mice were collected while the mice were anaesthetised with isoflurane. A lateral view, a dorsal-ventral view and a skull image were all taken to enable a full qualitative assessment of the integrity of the skeleton. A 2 cm lead bar was utilised to provide a calibration scale for the measurement of the tibia length.

### CSD analysis

The CSD test identifies physical and behavioural abnormalities through observation (Rogers et al., 1997). Mice were individually observed in a series of environments to test a range of attributes, including hearing, visual placement, activity, motor coordination, righting ability as well as morphological features.

### DEXA

The body composition of the mice was assessed using a PIXImus DEXA machine (GE Medical Systems, USA). Mice were anaesthetised with ketamine/xylazine and high-energy X-ray images were automatically analysed for fat tissue content, lean tissue content, bone mineral density and bone mineral content. While anaesthetised, the mice also underwent the auditory brainstem response test (see below).

### ECHO-MRI

Body composition of the mice was assessed using an EchoMRI^TM^ whole-body composition analyser (Echo Medical System, Houston, TX, USA). The analysis output quantified fat mass, lean mass and water content of the mice.

### Histopathology

Histopathology was performed on all major organs of four male and four female mice of each genotype (WT and Dp1Tyb) at the end of the cohort 1 pipeline. Following necropsy, heart, spleen and kidneys were weighed. These and other tissues were fixed in 10% neutral buffered formalin, wax embedded, sectioned and stained with Haematoxylin and Eosin. Slides were reviewed by two veterinary pathologists. Significant findings were scored using a non-linear semi-quantitative approach (Scudamore, 2014).

### Histology for Aβ

Immediately following euthanasia, the brain was removed and immersion fixed in 10% buffered formal saline (Pioneer Research Chemicals, UK). After 48-72 h, the brain was blocked into 3 mm coronal slices using an adult mouse brain matrix, and slices were embedded in paraffin wax using a Sakura VIP6 Automated Vacuum Tissue Processor. A series of 4 μm sections comprising the dorsal hippocampus were cut and mounted onto SuperFrost Plus glass slides. For Aβ immunostaining, sections were dewaxed, rehydrated through an alcohol series to water, pre-treated with 80% formic acid for 8 min, followed by washing in distilled water for 5 min. Sections were loaded as wet mounts into a Ventana Discovery XT automated stainer, where further pre-treatment for 30 min with mild CC1 (EDTA boric acid buffer, pH 9.0) and blocking for 8 min with Superblock (Medite, #88-4101-00) were performed prior to incubation for 8 h with biotinylated mouse monoclonal antibody 4G8 (2 μg/ml; Sigma-Aldrich SIG-39240 Beta-Amyloid). Staining was completed with a Ventana XT DABMap kit and a Haematoxylin counterstain, followed by dehydration and permanent mounting with DPX. All images were acquired using a Leica DM2000 LED microscope fitted with a MC190 HD camera.

### Calorimetry

The metabolic rate of the mice was assessed using indirect calorimetry. Mice were individually housed overnight for 21 h in Phenomaster cages (TSE Systems, Germany) with standard bedding and igloos. Oxygen consumption (VO_2_) and carbon dioxide production (VCO_2_) were simultaneously measured through an indirect gas calorimetry system air sampling, and from this the respiratory exchange ratio (VCO_2_/VO_2_) and heat production were calculated. Activity was monitored using a photobeam-based system from which speed of movement of the animal was calculated.

### Echocardiogram and ECG

The cardiac phenotype was assessed using both echocardiogram and ECG. For mice in cohort 1, these procedures were conducted at the same time. Mice were anaesthetised under isoflurane, and the ECG was recorded using BioAmp (AD Instruments, Australia) and LabChart Pro software (AD Instruments). For the echocardiogram, the left ventricle of the heart was imaged and analysed using the Vevo770 (Fujifilm Visualsonics, USA).

### Plethysmography

Mice were individually placed into whole-body plethysmography chambers (Data Sciences International) and allowed to acclimatise for 30 min. Breathing volumes of the mice were recorded initially for 5 min in room air (baseline, normoxia), then for 5 min in 10% O_2_/3% CO_2_ (hypoxia) and a further 5 min in room air (recovery, normoxia). Any mice experiencing breathing problems were removed from the chambers immediately.

### Auditory brainstem response

To determine their hearing range, mice were anaesthetised with ketamine/xylazine, and the auditory brainstem response was recorded in response to either a click sound or to tones at 8 kHz, 16 kHz and 32 kHz, using subdermal electrodes placed on the vertex and the left and right bulla (Hardisty-Hughes et al., 2010; Ingham et al., 2011). Intensity of the sounds was increased from 0 dB to 85 dB sound pressure level and the threshold of the response was determined as the lowest sound intensity that gave a recognisable auditory brainstem response waveform response.

### Opthalmoscopy

Eye morphology and visual response of the mice were assessed using a slit lamp and an opthalmascope. Tropicamide was used to dilate the pupils, and observations were manually scored for morphological or response abnormalities.

### Glucose tolerance test

Mice were fasted overnight for 18 h. A sample of blood was analysed from the tail, to determine the fasted blood glucose concentration using the Accu-Chek glucose meter (Abbott, UK). The mice were injected intraperitoneally with 20% glucose solution (2 g glucose/kg body weight). Blood glucose measurements were taken again at 15, 30, 60 and 120 min after injection of glucose.

### Blood collection

Mice were anaesthetised with isoflurane, and blood was collected under anaesthesia from the retro-orbital sinus into lithium heparin-coated tubes for clinical chemistry and EDTA-coated tubes for haematology. The mice were either free fed (cohort 1) or fasted overnight for 18 h (cohort 2).

### Clinical chemistry and ELISA

Lithium heparin samples from free-fed and fasted mice were kept on wet ice and centrifuged within 1 h of collection for 10 min at 5000 ***g*** in a refrigerated centrifuge at 8°C. The resulting plasma samples were frozen until analysis. Clinical chemistry of free-fed plasma was analysed with a Beckman Coulter AU680 clinical chemistry analyser using reagents and settings recommended by the manufacturer for the analysis of ALT, albumin, alkaline phosphatase, alpha-amylase, AST, calcium, chloride, creatinine kinase, free fatty acids, fructosamine, glucose, glycerol, high-density lipoprotein-cholesterol, inorganic phosphorus, iron, potassium, sodium, total bilirubin, total protein, triglycerides and urea. In addition, glucose and triglycerides were also measured in plasma from fasted mice. Insulin, glucagon, leptin and adiponectin were measured in plasma from fasted mice using ELISA kits from Millipore (EZRMI-13K), Mercodia (10-1281-01), Biovendor (RD291001200R) and R&D Systems (MRP300), respectively.

### Lipidomic analysis of plasma

To profile the lipidome, 15 µl plasma from either free-fed or fasted mice was analysed. Lipids were extracted using 250 µl methanol, sonicated and centrifuged, and the supernatant was dried under nitrogen. Extracts were reconstituted in 150 µl of 1/2 (v/v) methanol/water, and 2 µl was analysed using a Waters Xevo G2 Quadrupole Time of Flight (Q-ToF) mass spectrometer connected to a Waters Acquity ultra-performance liquid chromatogram (UPLC) (Milford, MA, USA). Chromatography was performed using a Waters Acquity UPLC CSH C18 1.7 µm, 100×2.1 mm column, a linear gradient consisting of solvent A [10 mM ammonium formate in 60/40 (v/v) acetonitrile/water] and solvent B [10 mM ammonium formate in 10/90 (v/v) acetonitrile/propan-2-ol] (flow rate, 0.4 ml/min; temp, 55°C; starting conditions 45% B, 7.5 min 98% B). Ions were detected in positive mode with a source temperature of 12°C, desolvation temperature of 550°C, capillary voltage of 1.5 kV, cone voltage of 30 V, cone gas flow of 50 l/h and desolvation gas flow of 900 l/h. Data were converted to netCDF format, and the matchedFilter peak-finding algorithm of the xcms software (Smith et al., 2006) was used to identify mass peaks. Annotation of lipids was based on exact mass using the LipidMaps database (https://lipidmaps.org/), fragmentation and chromatographic retention time. Data were processed using OPLS-DA within the SIMCA package (Umetrics, Umea, Sweden) and univariate statistics as described below.

### Haematology

Full blood counts and differential analyses of whole-blood samples collected from free-fed mice (cohort 1) in EDTA-coated tubes were performed with a Siemens Advia 2120 analyser using reagents and settings recommended by the manufacturer.

### Flow cytometry

Single-cell suspensions from spleen, bone marrow, thymus, mesenteric and peripheral lymph nodes, peritoneal cavity and blood were depleted of erythrocytes using ACK lysis buffer as previously described (Schweighoffer et al., 2013), before staining cells with a mixture of antibodies in PBS containing the live/dead marker Zombie Aqua (BioLegend). Antibodies against the following antigens were used [indicating antigen-fluorophore (clone)]: B220-BV605 (RA3-6B2), B220-FITC, B220-PE, CD3ε-PE (145-2c11), CD11b-FITC (M1/70), CD11c-PerCP/Cy5.5 (N418), CD19 APC (1D3), CD23-APC (B3B4), CD38-PE (90), CD42d-APC (1C2), CD62L-BV711 (MEL-14), CD49b-FITC (Dx5), CD115-PE (AFS98), CD138-PE/cy7 (281-2), CXCR5-BV785 (L138D7), Gr-1-PE (RB6-8C5), GR-1-FITC, Ly6C-BV711 (HK1.4), Ly6G-FITC (1A8), PD-1-PE/Cy7 (29F.1A12), TCRγδ-BV605 (GL3) and Thy1.2-BV605 (30-H12) from BioLegend; B220-APC/eF780, CD2-PE (RM2-5), CD3ε-FITC, CD4-APC (RM4-5), CD5-PE (53-7.3), CD8-FITC (53-6.7), CD8-PE, CD11b-eF450, CD21-APC/eF780 (4E3), CD25-PE (PC61.5), CD44-APC (IM7), CD71-PE (RI7 217.1.4), CD73-PE/Cy7 (eBioTY-11.8), CD93-APC (AA4.1), Fas-PE/Cy7 (Jo), F4/80-APC (BM8), IgD-eF450 (11-26), NK1.1-PE/Cy7 (PK136) and TCRβ-PerCP/Cy5.5 (H57-597) from eBioscience; CD41-FITC (MWReg30), Fas-PE-CF594, IgG1-APC (X56), PD-L2-BV421 (Ty25) and TER-119-BV421 (TER-119) from BD Biosciences. Goat-anti-mouse IgM Fab-FITC was purchased from Jackson ImmunoResearch. To exclude lineage^+^ cell populations, cells were stained with antibodies against B220, CD3ε, CD11b (also known as ITGAM) and Gr-1 for erythrocyte and megakaryoblast staining in spleen and bone marrow; B220 and CD3ε for myeloid cell staining in spleen and blood; and B220, CD4, CD8, TCRβ, TCRγδ, Gr-1, CD11b, CD11c (also known as ITGAX), CD49b (also known as ITGA2) and NK1.1 (also known as KLRB1C) for double negative thymocyte staining. An unlabelled antibody against CD16/32 (Fc-block, eBioscience) was used in all stainings to avoid non-specific binding of fluorophore-labelled antibodies to Fcγ receptors. Data were acquired on an LSRII flow cytometer (Becton Dickinson) and analysed using FlowJo v10.5 (TreeStar).

### Open field habituation

The open field habituation test assesses anxiety, activity and exploratory behaviour in first novel and then familiar environments. Mice were placed in well-lit arenas (150-200 lux, 44×44 cm) for 30 min, and the activity of the mice in the centre zone (40% of the total area), periphery (area 8 cm towards the centre from the wall) and whole arena was captured in 5 min bins using Ethovision software (version 8.5). Mice were returned to the arena on the following day and measurements repeated to assess habituation.

### Elevated zero maze

The elevated zero maze assesses the conflict between exploratory behaviour of novel environments and avoidance of well-lit open areas (Crawley, 1999) and uses an elevated maze (53 cm off the ground) in the shape of a circle which is divided equally into two open sections and two closed sections, with each section being ∼30 cm long. Mice were placed onto an open section and allowed to explore for 5 min. The video was analysed using Ethovision software, and the total amount of time spent in either the open or closed sections was determined.

### Acoustic startle and PPI

The acoustic pre-pulse startle test measures sensorimotor gating mechanisms through exposing the mice to a loud startle noise, which may or may not be preceded by a quieter tone of differing sound levels. As the intensity of the pre-pulse tone increases, the brain should increasingly disregard the following startle noise, resulting in decreased flinching. Mice were placed in an acoustic startle chamber (Med Associates Inc., USA) and acclimatised to a background noise level of 50 dB for 5 min. Mice were then exposed to a startle tone of white noise at 110 dB for 40 ms, either on its own or preceded 50 ms earlier by a 10 ms pre-pulse white noise at 55, 65 or 70 dB (PPI1, PPI2 or PPI3, respectively) in pseudorandom order. Responses to the startle tone were measured for 100 ms following the start of the startle tone using a piezoelectric transducer in the floor of the chamber, which detected movement of the animal. Each trial condition was tested ten times.

### Fear conditioning test

Fear conditioning assesses the memory of an aversive experience and determines whether it is based on the cue, the context or both (Crawley, 1999). Mice were placed into square chambers (17 cm^2^) on day 1 and allowed to explore for 150 s, during which the amount of freezing behaviour was measured (baseline). An auditory stimulus (70 dB) was presented for 30 s and was followed by one foot shock (0.5 mA, 0.5 s). After an interval, the auditory stimulus and foot shock were repeated twice more before the mouse was returned to its home cage. On day 2, the mice were placed into the same chambers, and the amount of freezing behaviour was measured over a 5 min period (context). Four hours later, the mice were placed into circular chambers (20 cm in diameter) with additional vanillin essence to reinforce the novel environment setting. Freezing in the new arena was measured for 180 s (pre-cue), after which the auditory stimulus was presented alone and again the amount of freezing behaviour was measured (post-cue). The comparison of baseline versus context or pre-cue versus post-cue percentage freezing behaviour was used to evaluate associative memory to context or cue, respectively.

### Object-in-place test

Mice were first habituated to a transparent Plexiglas arena (60×60×40 cm) for 10 min for two consecutive days. To test for object-in-place associations, mice were placed in the arena with three distinct objects for 10 min, returned to their home cage for 10 min and brought back into the arena for a further 10 min (sample phases 1 and 2). The mice were returned to their home cage for either 10 min or 3 h and then once again brought into the arena for 10 min, but this time the position of two of the objects in the arena had been exchanged (test phase). Behaviour of mice was recorded with an overhead video camera, and subsequently contact times of the mice with each object were determined; contact was defined by the mouse being ≤1 cm from the object and facing towards it. A discrimination ratio was calculated to reflect the preference for contact with the two switched objects compared to the object that had not been moved. The ratio ranged from 0 to 1; ratios over 0.5 indicated a preference for the switched objects and thus intact object-in-place associative memory.

### Sleep analysis

Analysis of sleep/wake cycles was performed using video tracking as previously described (Fisher et al., 2012). Briefly, mice were singly housed and placed in light-controlled chambers with near infra-red miniature CCD cameras positioned above the cages (Maplin, UK). Monitoring during dark periods was performed using infra-red illumination. Mice were allowed to acclimatise to the home cage for 24 h in a 12 h light/dark cycle (100 lux light intensity) before data collection. Video monitoring was then performed for a 24 h period in a 12:12 h light/dark cycle. Video files were uploaded to ANY-maze video analysis software (Stoelting, USA), which was used to track mouse mobility and to score cumulative periods during which the animals remained immobile for 40 s or more, a measure showing very high correlation with sleep (Fisher et al., 2012).

### Analysis of motor function

Mice were singly housed in cages equipped with modular running wheels (TSE Systems, Germany) (Mandillo et al., 2014). The average number of rotations, duration of each running bout and distance travelled every night were calculated using TSE Systems software over the course of 21 days. At the end of 14 days, the wheel was changed for a complex wheel in which a number of rungs had been removed, allowing assessment of the ability of the mice to adjust to a more difficult locomotor activity.

### Locotronic test

The Locotronic test (Intellibio, France) is a test of motor coordination capability. Mice traverse a horizontal ladder with evenly spaced rungs, along a narrow corridor to reach the exit. The number of rungs that the mouse stepped on or missed was recorded automatically; a missed rung was considered an error. Each animal was tested two to three times. Trials in which mice took more than 60 s to traverse the ladder were excluded from the analysis; a total of 11 out of 146 trials were excluded, five from WT mice and six from Dp1Tyb mice, indicating no difference in motivation between genotypes.

### Grip strength test

Grip strength was measured using a grip strength meter (Bioseb, France), recording the maximum force generated by a mouse using just its forelimbs or all four limbs. Grip strength measures were carried out in triplicate for each mouse.

### Statistical analysis

For each of the above procedures, we took all the phenotype measurements for the Dp1Tyb mice and compared these to values from WT control mice. Using fitted linear models, the null hypothesis was that a fitted model without a genotype term is as good a fit to the data as a model with the addition of a genotype term. The significance of the good fit is taken as the *P*-value for a genotype effect between the mutant and WT [p(genotype)]. In order to adjust for parameters that have non-normal distributions, a Box–Cox procedure (Box and Cox, 1964) was applied to the data prior to analysis. For continuous variables, a mixed model approach was used to compensate for any batch effects within the data. We constructed a linear-mixed-model of phenotype value as a function of genotype [‘value∼1+sex+experimentrepeat+(1|dop)+genotype’] and compared this with the model without a genotype term [‘value∼1+sex+experimentrepeat+(1|dop)’], where ‘dop’ is the date of procedure. A two-way ANOVA test was used to generate a p(genotype) value for the genotype effect. Where there were no repeated measurements for a parameter, or only a single sex was measured, the ‘sex’ and ‘experiment repeat’ formula terms were dropped from the models. For discrete variables, an analogous logistical regression model method was used with a model formula similar to the one used for the continuous method but removing the date of procedure mixed effect (as logistical regression does not support it). Once the p(genotype) value was determined for a parameter, the FDR adjustment (Storey, 2002) was used to generate FDR-adjusted *q*-values: q(genotype). A *q*-value of ≤0.05 was considered significant, and q(genotype) values are indicated on figures.

A comparison of variances between the WT and Dp1Tyb data for each parameter was carried out using Brown–Forsythe Levene-type test for equality of variances (Brown and Forsythe, 1974), which produces a *P*-value with the null hypothesis that each group has the same variance. FDR adjustment was then carried out on this set of *P*-values, to produce FDR-adjusted *q*-values for the similarity of variances: q(variance). Sexual dimorphism (sexdim) was assessed by using a mixed model comparing a model with sex-genotype interaction [‘value∼1+sex+experimentrepeat+(1|dop)+sex:genotype’] with a model without the interaction [‘value∼1+sex+experimentrepeat+(1|dop)+genotype’]. Two-ANOVA was used to determine the significance of the sexdim, and FDR was then applied to this set of *P*-values to produced *q*-values, q(sexdim). Metadata fields such as ‘experimenter id’ or ‘anaesthesia’ were analysed to see whether the metadata field had a significant effect to the phenotype calls. If the metadata field produced a significant effect on the model (two-ANOVA, *P*≤0.05), it was added as a factor to the linear model, compensating for its effect. Coefficient of variation for each parameter with continuous variables was calculated by dividing the standard deviation by the mean, treating each sex and genotype separately.

## Supplementary Material

Supplementary information
