# Supplementary material for: Comprehensive phenotypic analysis of the Dp1Tyb mouse strain reveals a broad range of Down syndrome-related phenotypes
Source: Dis Model Mech. 2021 Oct 18;14(10):dmm049157. doi: 10.1242/dmm.049157 (PMC8543064; doi:10.1242/dmm.049157)
Supplement: Supplementary information [file dmm-14-049157-s1.pdf]

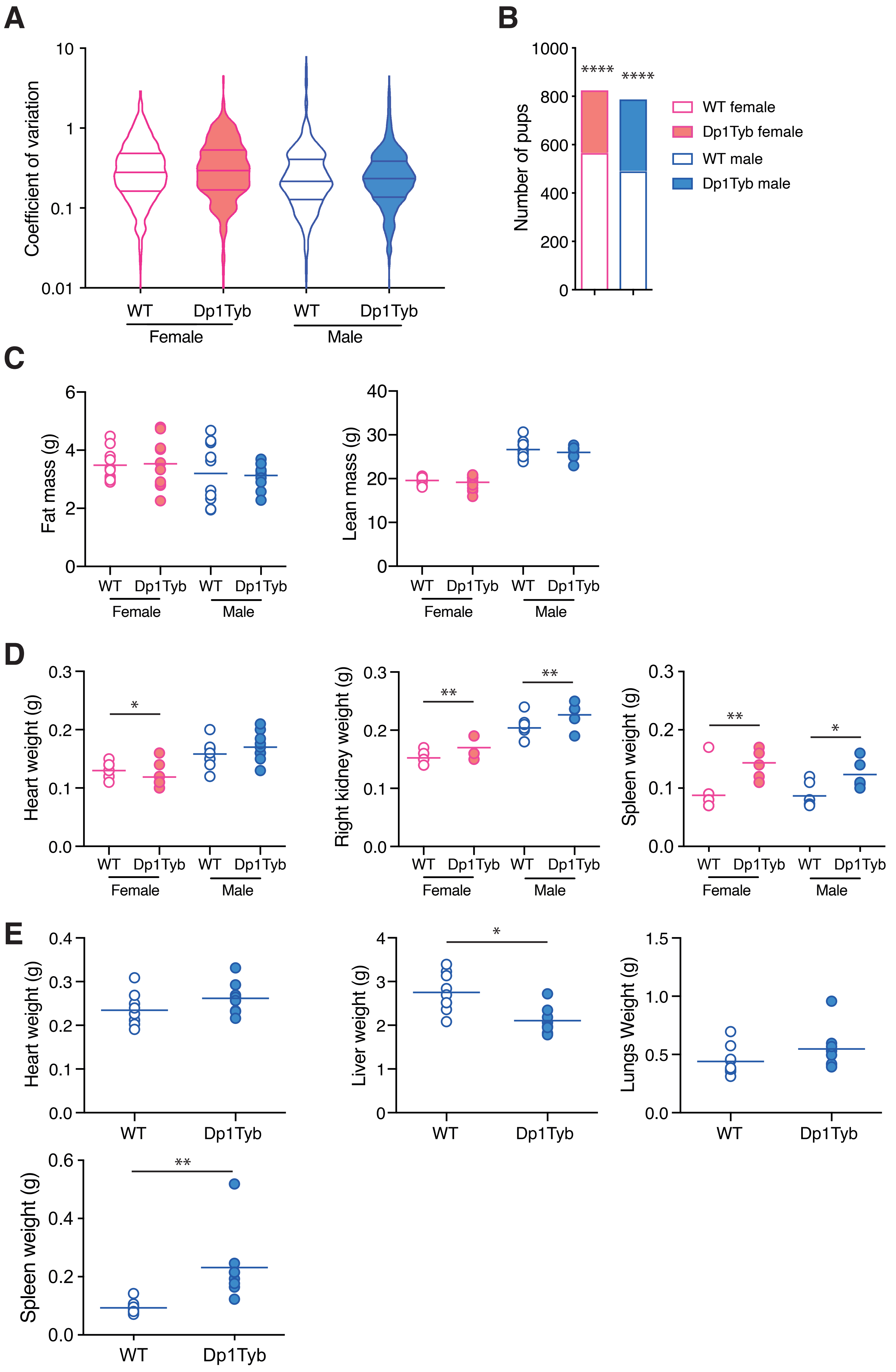


Disease Models & Mechanisms • Supplementary information

**Fig. S1. Decreased viability of Dp1Tyb mice. A**, Coefficient of variation (standard deviation/mean) for all numerical parameters analysed shown as violin plots, with horizontal lines indicating median and 25$^{th}$ and 75$^{th}$ centiles. There are no significant differences between WT and Dp1Tyb measurements. Note the medians differ between females and males because some tests were carried out on only one sex (e.g. flow cytometric analysis was only carried out on females). **B**, Number of WT and Dp1Tyb mice recovered at weaning. **C**, Fat mass and lean mass of WT and Dp1Tyb mice (cohort 1) determined using ECHO-MRI. **D, E**, Weights of organs determined at 16 (D) and 57 (E) weeks of age. Horizontal lines indicate mean. * 0.01 < *q* < 0.05; ** 0.001 < *q* < 0.01; **** *q* < 0.0001.

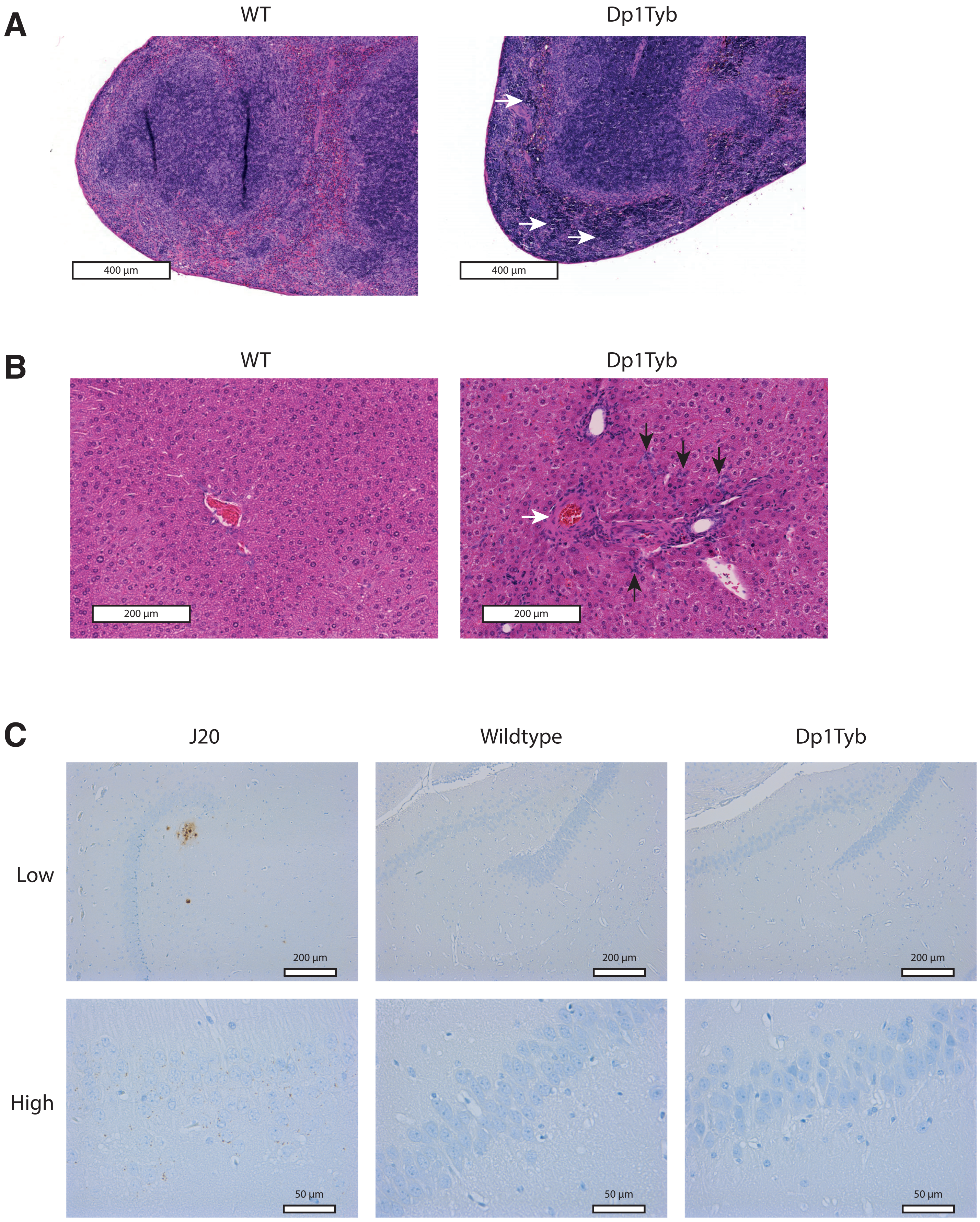


**Fig. S2. Pathological changes in spleens and livers of Dp1Tyb mice. A**, Sections of spleens from WT and Dp1Tyb mice (cohort 1) stained with haematoxylin and eosin showing increased extramedullary haematopoiesis in Dp1Tyb mice (white arrows). **B**, Sections of livers from WT and Dp1Tyb mice (cohort 1) stained with haematoxylin and eosin showing prominent vessels (white arrow) and bile duct hyperplasia in Dp1Tyb mice (black arrows). **C**, Immunohistochemistry staining of Aβ-positive extracellular plaques (brown) in the CA3/dentate gyrus region of the hippocampus of a 42-week old J20 mouse used as a positive control, and in 57-week old WT and Dp1Tyb mice. Images shown at low (10x) and high (40x) magnification. Intracellular Aβ-positive staining was evident in the J20 CA3 pyramidal neurons only; both WT and Dp1Tyb mice were negative for intracellular CA3 neuronal Aβ staining.

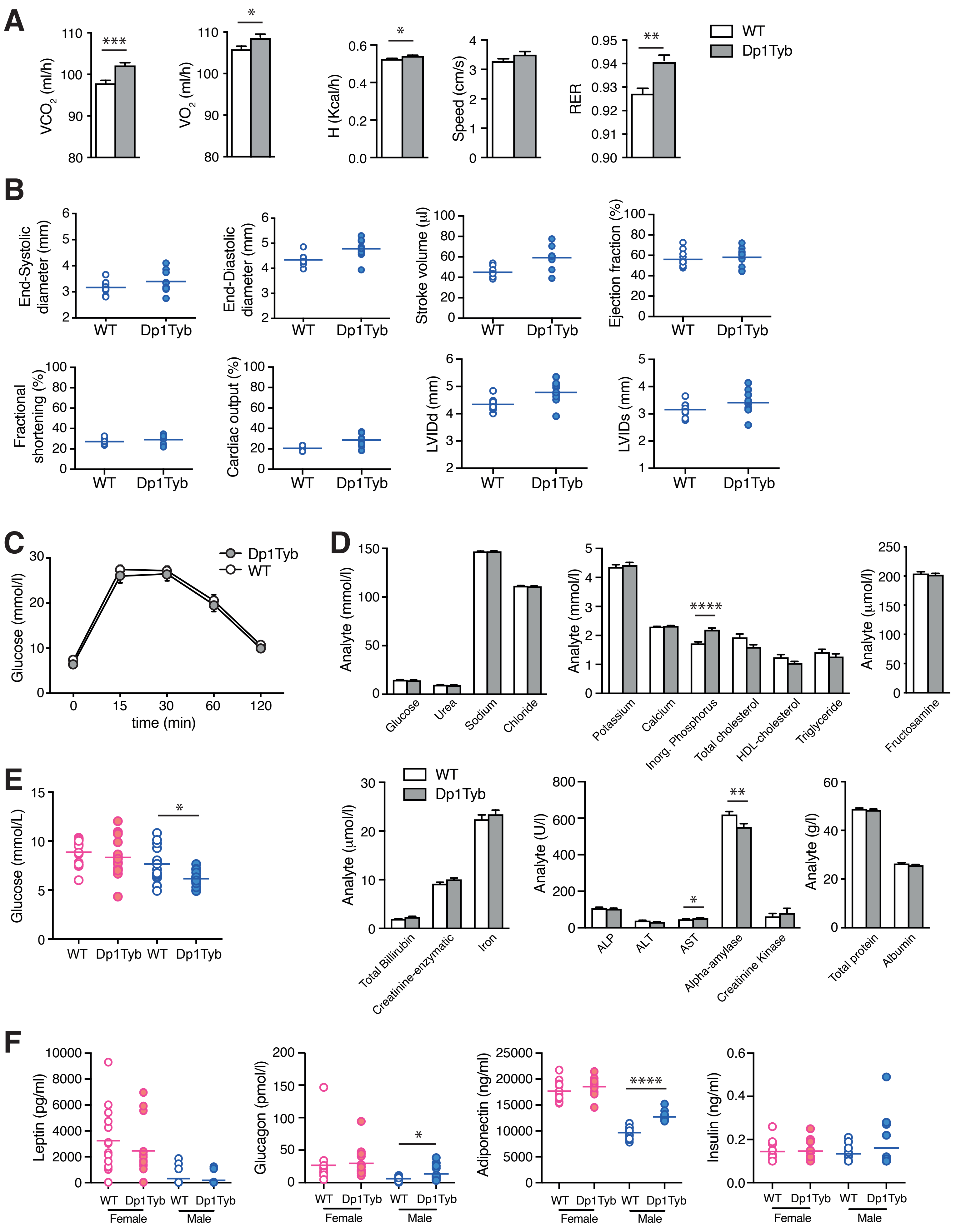


**Fig. S3. Calorimetry, echocardiography, glucose tolerance and plasma clinical chemistry of Dp1Tyb mice. A**, Mean±SEM $CO_2$ production ($VCO_2$), $O_2$ consumption ($VO_2$), heat production (H), movement speed and respiratory exchange ratio (RER) of WT and Dp1Tyb mice (cohort 1). **B**, End-systolic diameter, end-diastolic diameter, stroke volume, ejection fraction, fractional shortening, cardiac output, left ventricular inner diameter in diastole (LVIDd) and left ventricular inner diameter in systole (LVIDs) in WT and Dp1Tyb mice at 57 weeks of age (cohort 5) determined by echocardiography. Horizontal lines indicate mean. **C**, Mean±SEM blood glucose level in WT and Dp1Tyb mice (cohort 1) injected with 20% glucose solution. **D**, Mean±SEM concentrations of the indicated analytes in the blood of free-fed WT and Dp1Tyb mice (cohort 1). **E**, Levels of glucose in fasted WT and Dp1Tyb mice (cohort 2). **F**, Levels of the indicated hormones in the blood of fasted WT and Dp1Tyb mice (cohort 2). Horizontal lines indicate mean. * $0.01 < q < 0.05$; ** $0.001 < q < 0.01$; *** $0.0001 < q < 0.001$; **** $q < 0.0001$.

Disease Models & Mechanisms • Supplementary information

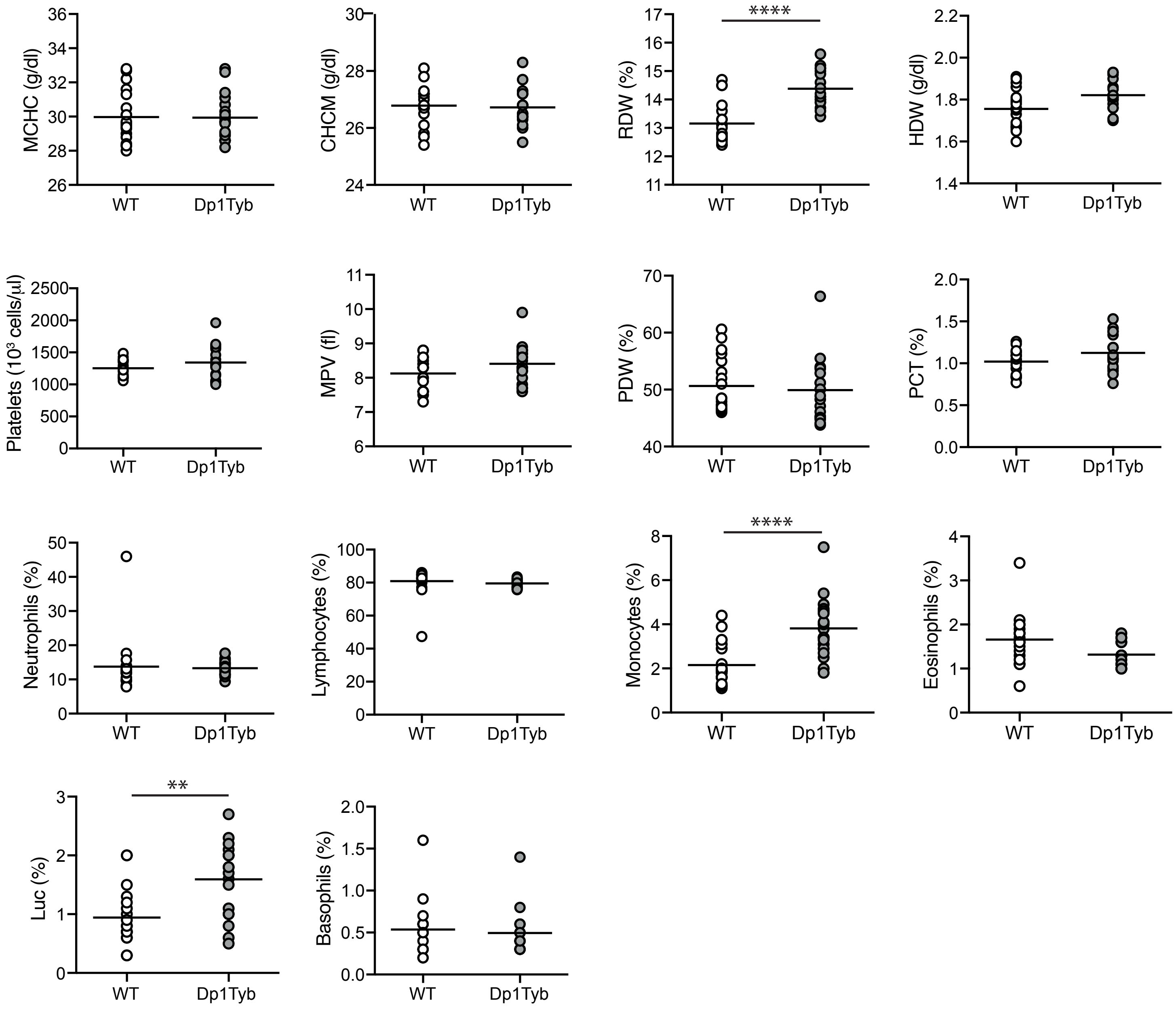


**Fig. S4. Haematology of Dp1Tyb mice.** Mean corpuscular haemoglobin concentration (MCHC), cell haemoglobin concentration mean (CHCM), red blood cell distribution width (RDW), haemoglobin distribution width (HDW), platelet concentration, mean platelet volume (MPV), platelet distribution width (PDW), plateletcrit (PCT), percentage of neutrophils, lymphocytes, monocytes, eosinophils, large unstained cells (Luc) and basophils in the blood of WT and Dp1Tyb mice (cohort 1). ** 0.001 < $q$ < 0.01; **** $q$ < 0.0001.

Disease Models & Mechanisms • Supplementary information

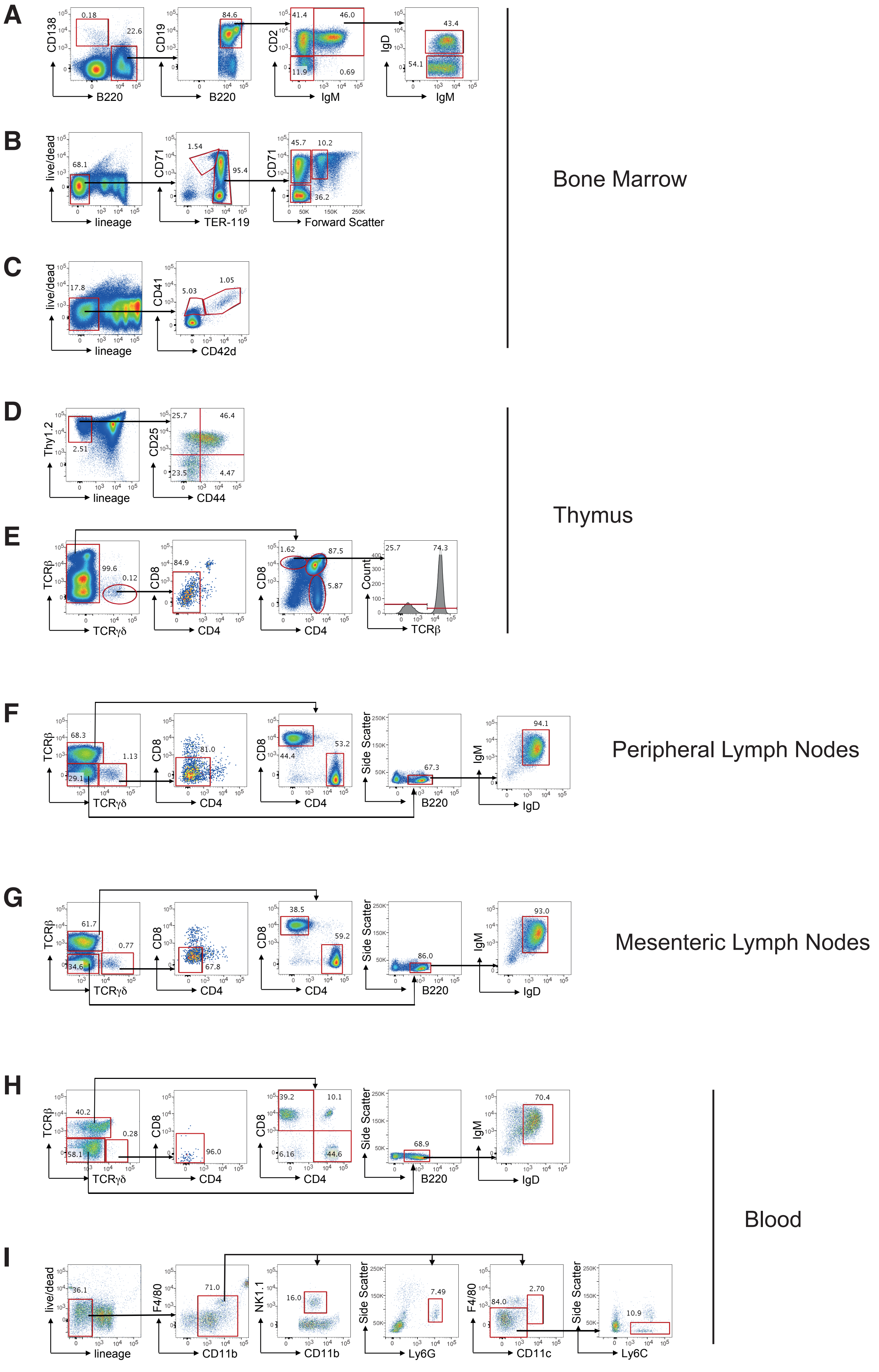


Disease Models & Mechanisms • Supplementary information

**Fig. S5. Flow cytometric gating strategies for analysis of bone marrow, thymus, lymph nodes, and blood.** Cell types were identified using the following gating strategies. **A-C**, Bone marrow. **A**, plasma cells ($B220^{-}CD138^{+}$), pro-B ($B220^{+}CD2^{-}IgM^{-}$), pre-B ($B220^{+}CD2^{+}IgM^{-}$), immature ($B220^{+}CD2^{+}IgM^{+}IgD^{-}$), mature ($B220^{+}CD2^{+}IgM^{+}IgD^{+}$) B cells. **B**, Lineage$^{-}$ cells ($B220^{-}CD3\varepsilon^{-}Mac\text{-}1^{-}Gr\text{-}1^{-}$) were subdivided into pro-erythroblasts (ProE, $CD71^{+}Ter119^{lo}$), EryA ($Ter119^{hi}CD71^{+}FSC^{hi}$), EryB ($Ter119^{hi}CD71^{+}FSC^{lo}$) and EryC ($Ter119^{hi}CD71^{-}FSC^{lo}$) erythroid progenitors. **C**, immature ($Lineage^{-}CD41^{+}CD42d^{-}$) and mature ($Lineage^{-}CD41^{+}CD42d^{+}$) megakaryoblasts. **D, E,** Thymus. **D**, Double negative (DN) thymocytes defined as Lineage$^{-}$ ($CD4^{-}CD8^{-}TCR\beta^{-}TCR\gamma\delta^{-}Gr\text{-}1^{-}CD11b^{-}CD11c^{-}Dx5^{-}Nk1.1^{-}B220^{-}$) and Thy1.2$^{+}$ were subdivided into DN1 ($CD44^{+}CD25^{-}$), DN2 ($CD44^{+}CD25^{+}$), DN3 ($CD44^{-}CD25^{+}$) and DN4 ($CD44^{-}CD25^{-}$). **E**, TCR$\gamma\delta$ thymocytes ($TCR\beta^{-}TCR\gamma\delta^{+}CD4^{-}CD8^{-}$), TCR$\gamma\delta^{-}$ cells were subdivided into double positive (DP, $CD4^{+}CD8^{+}$), CD4$^{+}$ single positives (SP, $CD4^{+}CD8^{-}$), CD8$^{+}$ intermediate single positives (ISP, $CD4^{-}CD8^{+}TCR\beta^{-}$), and CD8$^{+}$ SP ($CD4^{-}CD8^{+}TCR\beta^{+}$). **F-H,** Peripheral (F) and mesenteric (G) lymph nodes and blood (H). $\gamma\delta$ T cells ($TCR\beta^{-}TCR\gamma\delta^{+}CD4^{-}CD8^{-}$), TCR$\gamma\delta^{-}$ cells were subdivided into CD4$^{+}$ ($TCR\beta^{+}CD4^{+}CD8^{-}$) and CD8$^{+}$ ($TCR\beta^{+}CD4^{-}CD8^{+}$) T cells, B cells ($TCR\beta^{-}TCR\gamma\delta^{-}B220^{+}IgM^{+}IgD^{+}$). **I,** Blood. Lineage$^{-}$ cells ($B220^{-}CD4^{-}CD8^{-}$) were subdivided into NK cells ($CD11b^{lo}F4/80^{-}NK1.1^{+}$), neutrophils ($CD11b^{lo}F4/80^{-}Ly6G^{+}SSC^{hi}$), dendritic cells ($CD11b^{lo}F4/80^{-}CD11c^{+}$) and monocytes ($CD11b^{lo}F4/80^{-}CD11c^{-}Ly6C^{+}$). Numbers show percentage of cells falling into indicated gates.

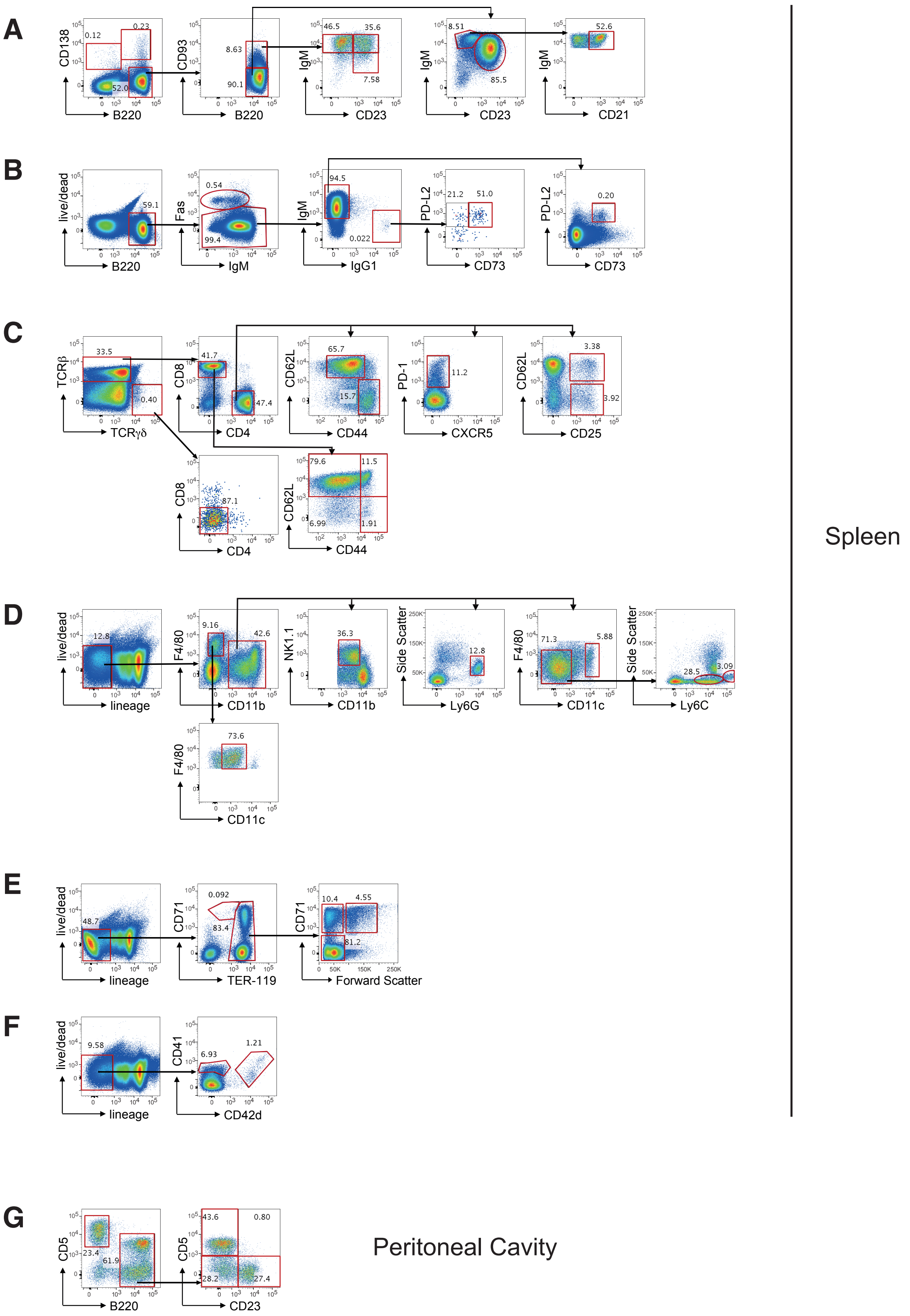


Disease Models & Mechanisms • Supplementary information

**Fig. S6. Flow cytometric gating strategies for analysis of spleen and peritoneal cavity.** Cell types were identified using the following gating strategies. **A-F,** Spleen. **A**, plasmablasts (B220$^{+}$CD138$^{+}$), plasma cells (B220$^{-}$CD138$^{+}$), transitional B cells (B220$^{+}$CD93$^{+}$) were subdivided into type 1 (T1, IgM$^{+}$CD23$^{-}$), T2 (IgM$^{+}$CD23$^{+}$) and T3 (IgM$^{lo}$CD23$^{+}$), mature B cells (B220$^{+}$CD93$^{-}$) were subdivided into follicular (IgM$^{+}$CD23$_{+}$) and marginal zone (IgM$^{+}$CD23$^{-}$CD21$^{+}$) B cells. **B**, Germinal centre (GC) B cells (B220$^{+}$Fas$^{+}$), IgM memory B cells (MBC) (B220$^{+}$Fas$^{-}$IgM$^{+}$IgG1$^{-}$CD73$^{+}$PD-L2$^{+}$) and IgG1 MBC (B220$^{+}$Fas$^{-}$IgM$^{-}$IgG1$^{+}$CD73$^{+}$PD-L2$^{+}$). **C**, $\gamma\delta$ T cells (TCR$\beta^{-}$TCR$\gamma\delta^{+}$CD4$^{-}$CD8$^{-}$), TCR$\beta^{+}$TCR$\gamma\delta^{-}$ cells were subdivided into total (CD4$^{+}$), naïve (CD4$^{+}$CD44$^{lo}$CD62L$^{+}$) and effector (CD4$^{+}$CD44$^{hi}$CD62L$^{-}$) CD4$^{+}$ T cells, T follicular helper T cells (CD4$^{+}$PD-1$^{+}$), regulatory T cells (CD4$^{+}$CD25$^{+}$CD62L$^{+\text{ or }-}$), total (CD8$^{+}$), naïve (CD8$^{+}$CD44$^{-}$CD62L$^{+}$), central memory (CD8$^{+}$CD44$^{+}$CD62L$^{+}$) and effector memory (CD8$^{+}$CD44$^{+}$CD62L$^{-}$) CD8$^{+}$ T cells. **D**, Lineage$^{-}$ cells (B220$^{-}$CD3$\varepsilon^{-}$) were subdivided into macrophages (CD11b$^{-}$CD11c$^{+}$F4/80$^{+}$), NK (CD11b$^{lo}$F4/80$^{-}$NK1.1$^{+}$), neutrophils (CD11b$^{+}$F4/80$^{-}$Ly6G$^{+}$SSC$^{hi}$), dendritic cells (CD11b$^{+}$F4/80$^{-}$CD11c$^{+}$) and monocytes (CD11b$^{+}$F4/80$^{-}$CD11c$^{-}$) subdivided into Ly6C$^{lo}$ and Ly6C$^{hi}$ cells. **E**, Lineage$^{-}$ cells (B220$^{-}$CD3$\varepsilon^{-}$Mac-1$^{-}$Gr-1$^{-}$) were subdivided into pro-erythroblasts (ProE, CD71$^{+}$Ter119$^{lo}$), EryA (Ter119$^{hi}$CD71$^{+}$FSC$^{hi}$), EryB (Ter119$^{hi}$CD71$^{+}$FSC$^{lo}$) and EryC (Ter119$^{hi}$CD71$^{-}$FSC$^{lo}$) erythroid progenitors. **F**, immature (Lineage$^{-}$CD41$^{+}$CD42d$^{-}$) and mature (Lineage$^{-}$CD41$^{+}$CD42d$^{+}$) megakaryoblasts. **G**, Peritoneal cavity. B1a (B220$^{+}$CD5$^{+}$CD23$^{-}$), B1b (B220$^{+}$CD5$^{-}$CD23$^{-}$) and B2 (B220$^{+}$CD5$^{-}$CD23$^{+}$) B cells and T cells (B220$^{-}$CD5$^{+}$). Numbers show percentage of cells falling into indicated gates.

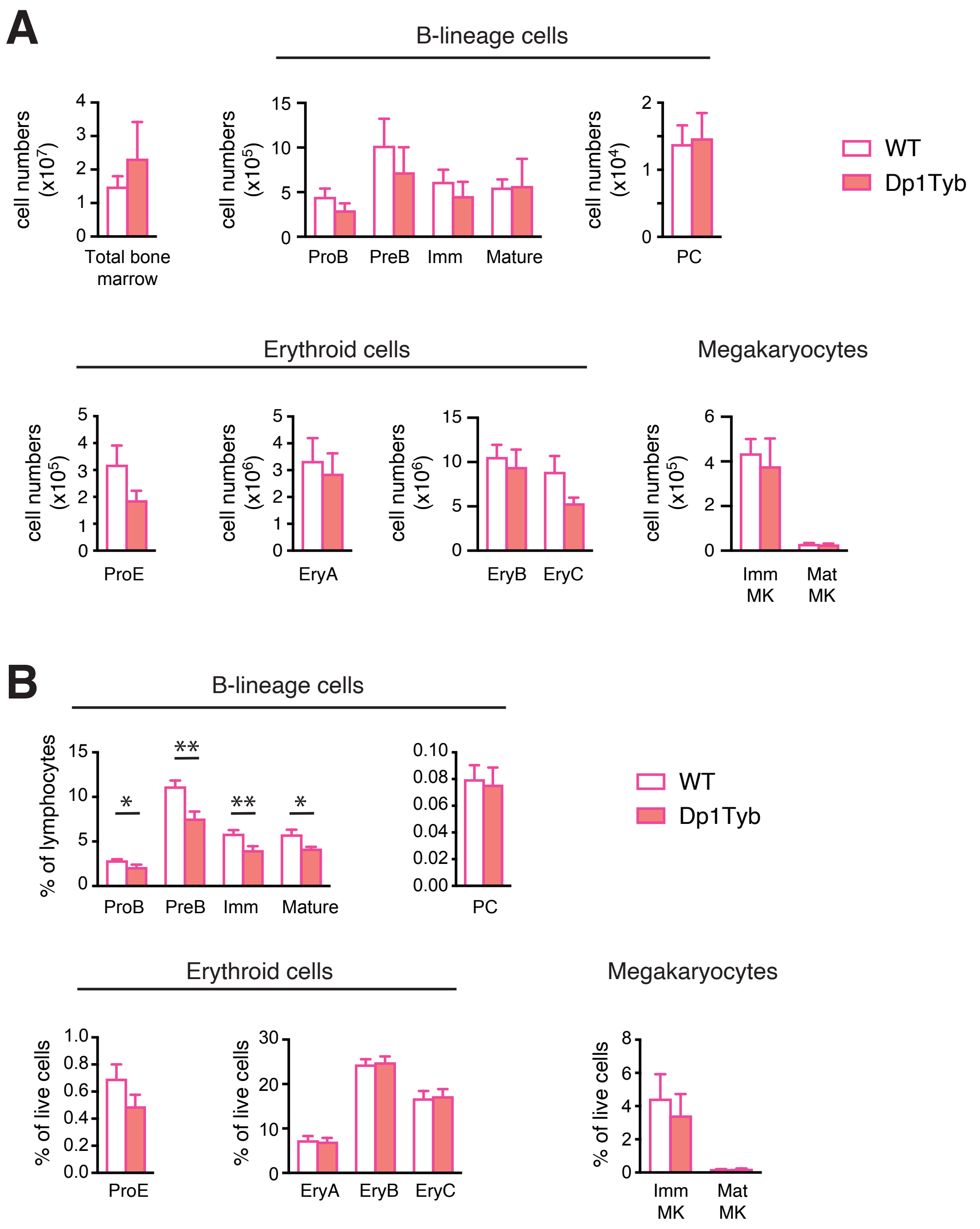


**Fig. S7. Flow cytometric analysis of bone marrow cells in Dp1Tyb mice. A**, Mean±SEM number of cells in the bone marrow of WT and Dp1Tyb mice (cohort 4) (2 tibia and 2 femurs/mouse), showing total cells, pro-B, pre-B, immature (Imm) and mature B cells, plasma cells (PC), pro-erythroblasts (ProE), EryA, EryB and EryC erythroid progenitors, and immature (Imm) and mature (Mat) megakaryoblasts (MK). **B**, Mean±SEM percentages of the populations shown in A. * 0.01 < $q$ < 0.05; ** 0.001 < $q$ < 0.01.

Disease Models & Mechanisms • Supplementary information

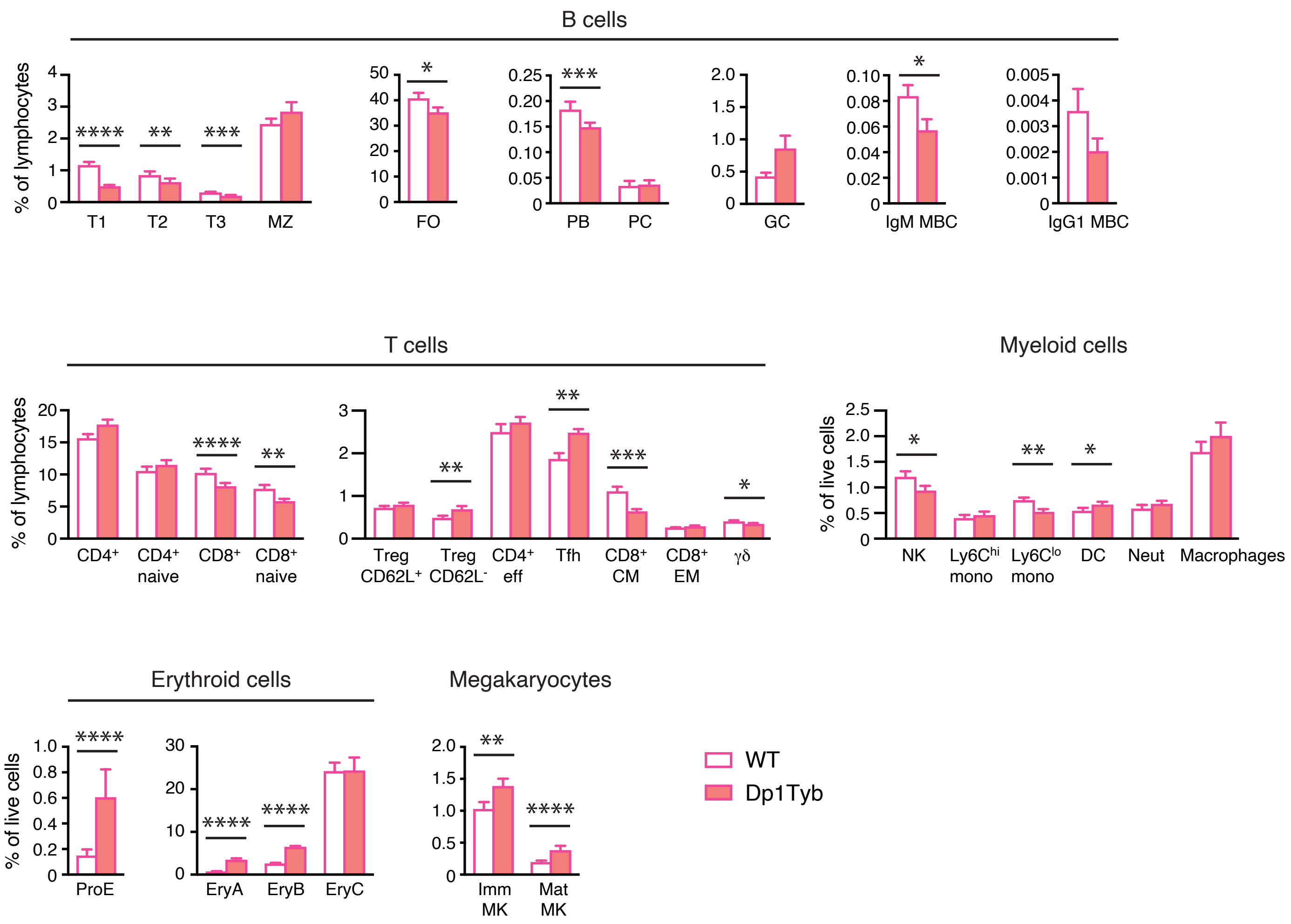


**Fig. S8. Flow cytometric analysis of splenocytes in Dp1Tyb mice.** Mean±SEM percentages of transitional type 1 (T1), T2, T3, marginal zone (MZ), follicular (FO), germinal centre (GC) B cells, plasmablasts (PB), plasma cells (PC), IgM and IgG1 memory B cells (MBC), and total or naive $CD4^+$ or $CD8^+$ T cells, $CD62L^+$ or $CD62L^-$ regulatory T cells (Treg), $CD4^+$ effector (eff) T cells, T follicular helper (Tfh) cells, $CD8^+$ central memory (CM) and effector memory (EM) T cells, $\gamma\delta$ T cells, NK cells, $Ly6C^{hi}$ and $Ly6C^{lo}$ monocytes (mono), dendritic cells (DC), neutrophils (Neut), macrophages, pro-erythroblasts (ProE), EryA, EryB and EryC erythroid progenitors, and immature (Imm) and mature (Mat) megakaryoblasts (MK) in the spleen of WT and Dp1Tyb mice (cohort 4). * $0.01 < q < 0.05$; ** $0.001 < q < 0.01$; *** $0.0001 < q < 0.001$; **** $q < 0.0001$.

Disease Models & Mechanisms • Supplementary information

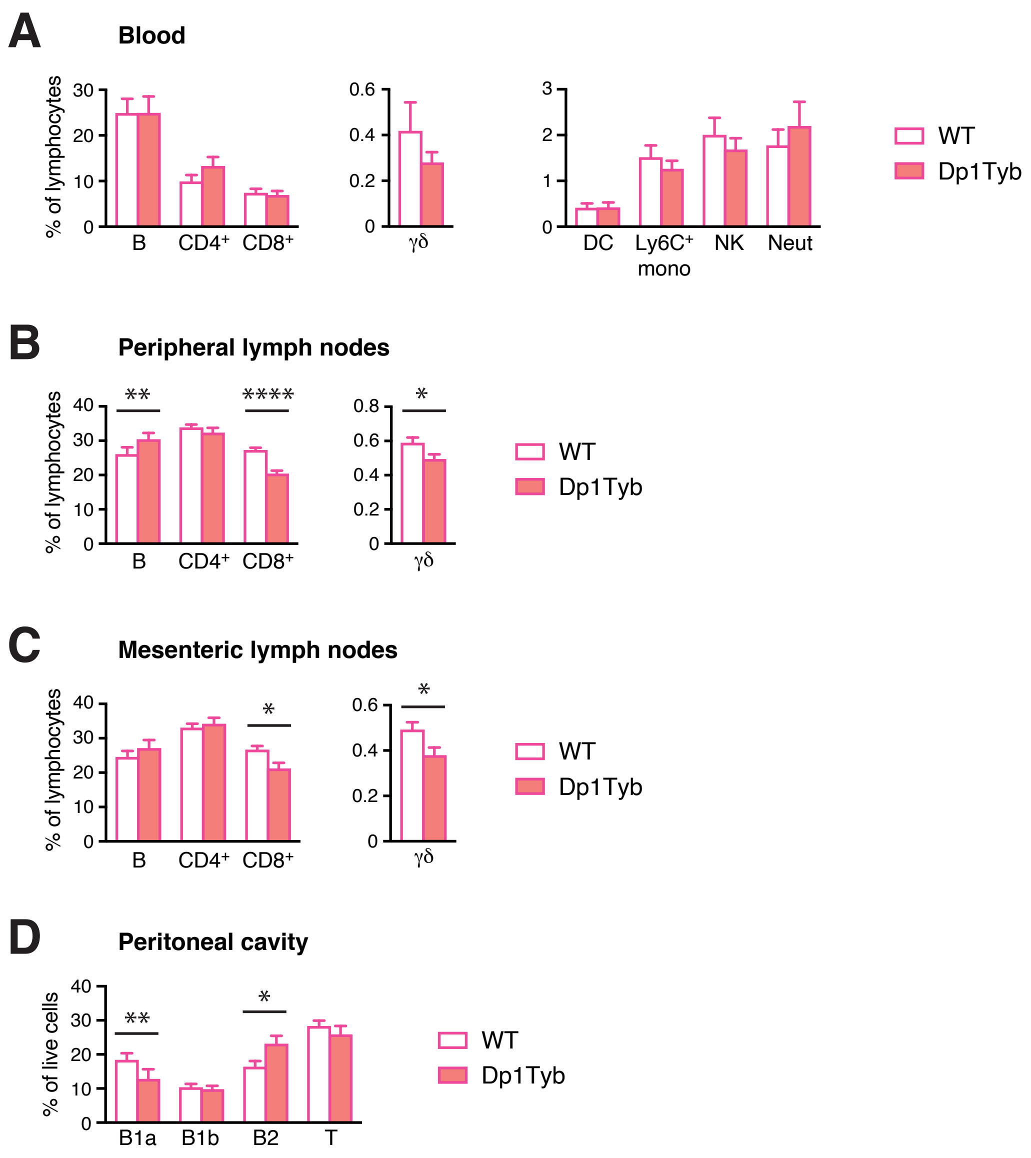


**Fig. S9. Flow cytometric analysis of blood, lymph nodes and peritoneal cavity in Dp1Tyb mice. A-C,** (**A**) Mean±SEM percentages of B cells, CD4$^+$, CD8$^+$ and $\gamma\delta$ T cells, dendritic cells (DC), Ly6C$^+$ monocytes (mono), NK cells, and neutrophils (Neut) in the blood, (**B**) peripheral lymph nodes, and (**C**) mesenteric lymph nodes of WT and Dp1Tyb mice (cohort 4). **D**, Mean±SEM percentages of B1a, B1b and B2 B cells and T cells in the peritoneal cavity of WT and Dp1Tyb mice (cohort 4). * $0.01 < q < 0.05$; ** $0.001 < q < 0.01$; **** $q < 0.0001$.

Disease Models & Mechanisms • Supplementary information

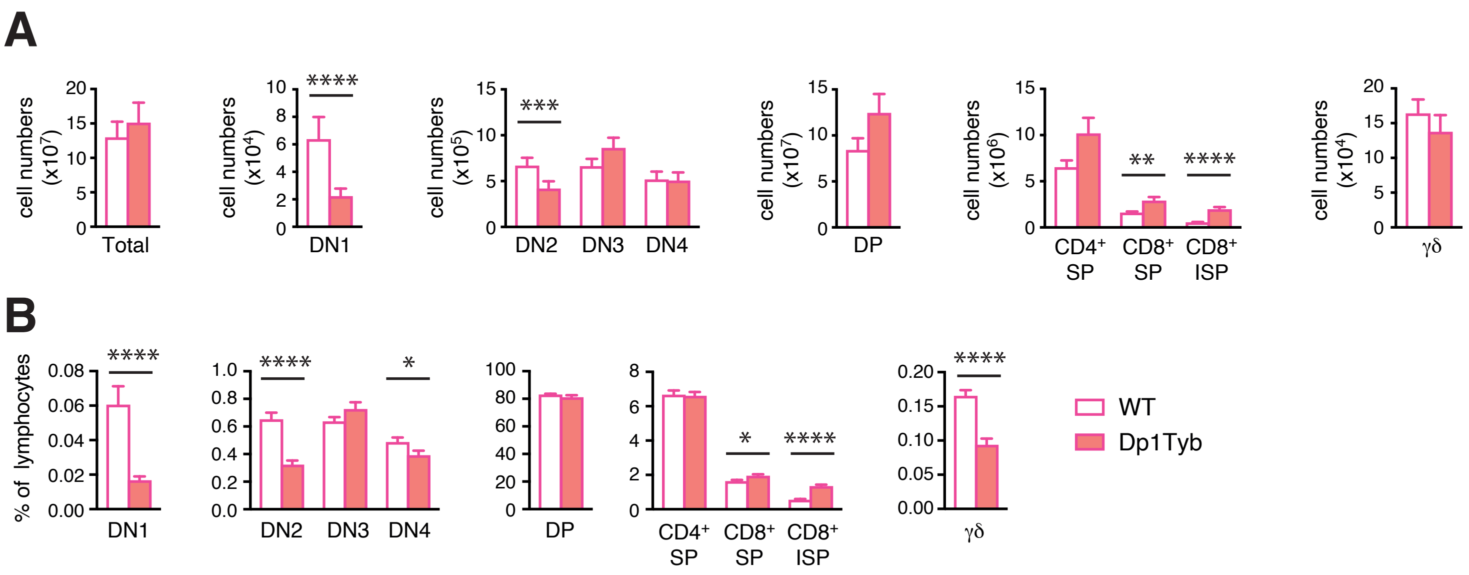


**Fig. S10. Flow cytometric analysis of thymocytes in Dp1Tyb mice. A**, Mean±SEM number of cells in the thymus of WT and Dp1Tyb mice (cohort 4), showing total cells, DN1, DN2, DN3, DN4, DP, $CD4^+SP$, $CD8^+SP$, $CD8^+ISP$ and $TCR\gamma\delta^+$ thymocytes. **B**, Mean±SEM percentages of the populations shown in A. * $0.01 < q < 0.05$; ** $0.001 < q < 0.01$; *** $0.0001 < q < 0.001$; **** $q < 0.0001$.

Disease Models & Mechanisms • Supplementary information

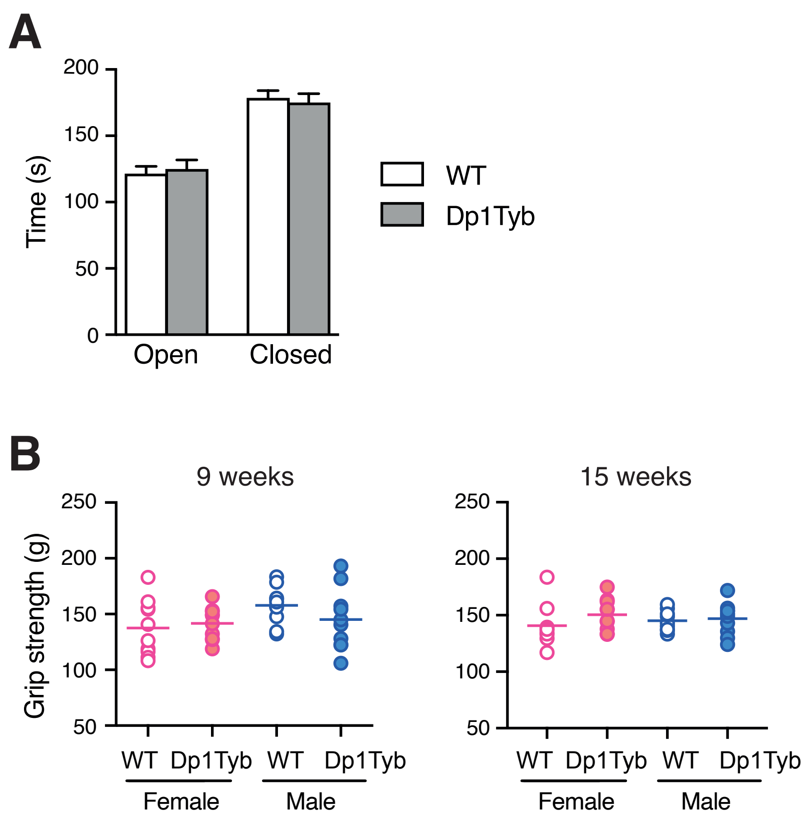


**Fig. S11. Elevated zero maze and grip strength tests.**

**A**, Mean±SEM time spent in the open and closed arms of an elevated zero maze by WT and Dp1Tyb mice (cohort 3). **B**, Grip strength of WT and Dp1Tyb mice (cohort 1) determined using all four limbs at 9 and 15 weeks of age. Horizontal lines indicate mean.

**Table S1. Statistical Analysis.**

Click here to download Table S1

**Table S2. Dp1Tyb mice have abnormal heads, snouts, lips and gait. A,** Table shows numbers of mice that gave the expected or not expected result in each of the tests in the Combined SHIRPA and Dysmorphology (CSD) tests. Dp1Tyb and WT mice were tested at 9 and 15 weeks of age (cohort 1). Test showing statistically significant differences (q<0.05) between Dp1Tyb and WT mice at either 9 or 15 weeks or both are highlighted in yellow. **B,** Locomotor activity measured as mean±sem number of squares entered by the mouse in 30s.

**A**

| | 9 weeks | | | | 15 weeks | | | | |
|---|---|---|---|---|---|---|---|---|---|
| | WT | | Dp1Tyb | | WT | | Dp1Tyb | | |
| **Test** | **As expected** | **Not as expected** | **As expected** | **Not as expected** | **As expected** | **Not as expected** | **As expected** | **Not as expected** | **Comments** |
| **Tail** | 19 | 1 | 14 | 6 | 17 | 4 | 10 | 10 | |
| **Coat** | 20 | 0 | 20 | 0 | 21 | 0 | 20 | 0 | |
| **Forelimbs** | 20 | 0 | 20 | 0 | 21 | 0 | 20 | 0 | |
| **Hindlimbs** | 20 | 0 | 20 | 0 | 21 | 0 | 20 | 0 | |
| **Vibrissae** | 19 | 1 | 20 | 0 | 21 | 0 | 20 | 0 | |
| **Head** | 18 | 2 | 11 | 9 | 19 | 2 | 8 | 12 | Abnormal shape |
| **Ears** | 20 | 0 | 20 | 0 | 21 | 0 | 20 | 0 | |
| **Snout Size** | 20 | 0 | 9 | 11 | 20 | 1 | 13 | 7 | Decreased |
| **Activity (body position)** | 19 | 1 | 17 | 3 | 21 | 0 | 18 | 2 | |
| **Tremor** | 20 | 0 | 17 | 3 | 3 | 18 | 14 | 6 | Tremor present |
| **Faecal pellet** | 19 | 0 | 20 | 0 | 21 | 0 | 20 | 0 | |
| **Feet position in jar** | 20 | 0 | 16 | 4 | 21 | 0 | 20 | 0 | |
| **Transfer Arousal** | 20 | 0 | 18 | 2 | 21 | 0 | 18 | 2 | |
| **Gait** | 20 | 0 | 14 | 6 | 20 | 1 | 6 | 14 | Lack of fluid movement |
| **Tail Elevation** | 20 | 0 | 20 | 0 | 21 | 0 | 20 | 0 | |
| **Startle Response** | 10 | 0 | 9 | 1 | 20 | 1 | 14 | 6 | |
| **Trunk Curl** | 20 | 0 | 19 | 1 | 21 | 0 | 20 | 0 | |
| **Limb Grasp** | 20 | 0 | 17 | 3 | 21 | 0 | 19 | 1 | |
| **Visual Placing** | 10 | 0 | 20 | 0 | 21 | 0 | 20 | 0 | |
| **Body Tone** | 20 | 0 | 20 | 0 | 21 | 0 | 17 | 3 | |
| **Limb Tone** | 20 | 0 | 17 | 0 | 16 | 5 | 14 | 6 | |
| **Paws** | 20 | 0 | 20 | 0 | 21 | 0 | 20 | 0 | |
| **Digits** | 20 | 0 | 20 | 0 | 21 | 0 | 20 | 0 | |
| **Nail** | 17 | 3 | 20 | 0 | 21 | 0 | 20 | 0 | |
| **Skin** | 20 | 0 | 20 | 0 | 21 | 0 | 20 | 0 | |
| **Teeth** | 20 | 0 | 20 | 0 | 21 | 0 | 20 | 0 | |
| **Genitalia** | 20 | 0 | 20 | 0 | 21 | 0 | 20 | 0 | |
| **Mouth Morphology** | 20 | 0 | 18 | 2 | 21 | 0 | 20 | 0 | |
| **Lip Morphology** | 18 | 2 | 6 | 14 | 21 | 0 | 19 | 1 | Abnormal shape |
| **Salivation** | 20 | 0 | 20 | 0 | 21 | 0 | 20 | 0 | |
| **Contact Righting** | 20 | 0 | 20 | 0 | 20 | 1 | 20 | 0 | |
| **Negative Geotaxis** | 20 | 0 | 16 | 4 | 21 | 0 | 17 | 3 | |
| **Vocalization** | 18 | 2 | 20 | 0 | 21 | 0 | 20 | 0 | |
| **Aggression** | 17 | 3 | 20 | 0 | 20 | 1 | 20 | 0 | |
| **Head Bobbing** | 20 | 0 | 20 | 0 | 21 | 0 | 20 | 0 | |

**B**

| | 9 weeks | | 15 weeks | |
|---|---|---|---|---|
| | **WT** | **Dp1Tyb** | **WT** | **Dp1Tyb** |
| **Locomotor Activity (Mean±sem)** | 26.55±1.07 | 30.15±1.86 | 20.45±1.23 | 22.38±2.53 |

Disease Models & Mechanisms • Supplementary information

**Table S3. Dp1Tyb mice have abnormally shaped heads.** Table shows numbers of Dp1Tyb and WT mice that were assessed as being normal or abnormal for each of the features listed from X-ray analysis of mice tested at 14 weeks of age (cohort 1). Test showing statistically significant differences (q<0.05) between Dp1Tyb and WT mice is highlighted in yellow.

| | **WT** | | **Dp1Tyb** | | |
|---|---|---|---|---|---|
| | **Normal** | **Abnormal** | **Normal** | **Abnormal** | **Comments** |
| **Skull shape** | 19 | 1 | 0 | 20 | Abnormal shape |
| **Zygomatic bone** | 19 | 1 | 19 | 1 | |
| **Maxilla** | 19 | 1 | 13 | 7 | |
| **Clavicle** | 20 | 0 | 20 | 0 | |
| **Scapulae** | 20 | 0 | 20 | 0 | |
| **Humerus shape** | 20 | 0 | 20 | 0 | |
| **Fusion of ribs** | 20 | 0 | 20 | 0 | |
| **Shape of ribs** | 20 | 0 | 20 | 0 | |
| **Vertebrae shape** | 19 | 0 | 19 | 0 | |
| **Fused vertebrae** | 19 | 1 | 19 | 1 | |
| **Processes on vertebrae** | 20 | 0 | 20 | 0 | |
| **Joints** | 20 | 0 | 20 | 0 | |
| **Syndactylism** | 20 | 0 | 20 | 0 | |
| **Brachydactyly** | 20 | 0 | 20 | 0 | |
| **Digit integrity** | 20 | 0 | 20 | 0 | |
| **Pelvis** | 18 | 2 | 16 | 4 | |
| **Femur shape** | 20 | 0 | 20 | 0 | |
| **Fibula** | 20 | 0 | 20 | 0 | |
| **Tibia** | 20 | 0 | 20 | 0 | |
| **Radius** | 20 | 0 | 20 | 0 | |
| **Ulna** | 20 | 0 | 20 | 0 | |
| **Mandibles** | 19 | 1 | 20 | 0 | |
| **Teeth** | 19 | 1 | 19 | 0 | |

Disease Models & Mechanisms • Supplementary information

**Table S4. Dp1Tyb mice have increased splenic extramedullary haematopoiesis, portal tract anomalies in the liver and otitis media.** Table shows incidence of tissues with an appearance considered microscopically within normal limits and tissues where pathological findings are present. Pathological findings may be spontaneous background lesions expected in the strain of mouse or considered specific to the genotype of the animals. Pathological analysis on WT and Dp1Tyb mice in cohort 1 was carried out at 16 weeks of age. Significant findings (different from variations in spontaneous C57BL/6J background pathology) in Dp1Tyb mice were increased splenic extramedullary haematopoiesis, portal tract anomalies in the liver and otitis media. These are highlighted in yellow.

| | Females | | | | Males | | | | |
|---|---|---|---|---|---|---|---|---|---|
| | WT | | Dp1Tyb | | WT | | Dp1Tyb | | |
| Tissue | Within normal limits | Pathological findings present | Within normal limits | Pathological findings present | Within normal limits | Pathological findings present | Within normal limits | Pathological findings present | Comments |
| Salivary gland | 4 | 0 | 3 | 1 | 2 | 2 | 5 | 0 | |
| Lungs | 2 | 2 | 1 | 3 | 4 | 0 | 2 | 3 | Increased perivascular inflammation in Dp1Tyb mice but considered within normal variation in background mouse strain. |
| Thyroid | 4 | 0 | 4 | 0 | 4 | 0 | 5 | 0 | |
| Trachea | 4 | 0 | 4 | 0 | 4 | 0 | 4 | 1 | |
| Parathyroid | 2 | 0 | 2 | 0 | 3 | 0 | 2 | 0 | |
| Kidney | 0 | 4 | 0 | 4 | 2 | 2 | 0 | 5 | Pelvic dilatation and inflammatory cell infiltrates seen in both genotypes. Significance uncertain but considered within normal variation in background mouse strain. |
| Heart | 4 | 0 | 4 | 0 | 4 | 0 | 5 | 0 | |
| Tongue | 4 | 0 | 4 | 0 | 4 | 0 | 5 | 0 | |
| Adrenal | 1 | 3 | 1 | 3 | 3 | 1 | 2 | 3 | Subcapsular cell hyperplasia in both genotypes but considered within normal variation in background mouse strain. |
| Liver | 0 | 4 | 0 | 7 | 3 | 1 | 0 | 4 | Significant lesion. Bile duct hyperplasia and prominent portal vasculature increased in Dp1Tyb mice. Not seen spontaneously. |
| Pancreas | 4 | 0 | 5 | 2 | 4 | 0 | 3 | 1 | Fatty atrophy and inflammatory cell foci in Dp1Tyb mice but considered within normal variation in background mouse strain. |
| Gall bladder | 3 | 0 | 6 | 0 | 3 | 1 | 2 | 2 | |
| Spleen | 0 | 4 | 0 | 7 | 0 | 4 | 0 | 4 | Significant lesion. Extramedullary haematopoiesis increased in Dp1Tyb mice. |
| Thymus | 3 | 1 | 5 | 2 | 4 | 0 | 3 | 1 | |
| Mesenteric lymph node | 3 | 0 | 2 | 0 | 3 | 0 | 3 | 0 | |
| Oesophagus | 3 | 1 | 4 | 0 | 4 | 0 | 4 | 0 | |
| Duodenum | 3 | 0 | 3 | 0 | 4 | 0 | 4 | 0 | |
| Jejunum | 4 | 0 | 4 | 0 | 4 | 0 | 4 | 0 | |
| Ileum | 4 | 0 | 4 | 0 | 4 | 0 | 4 | 0 | |
| Colon | 3 | 1 | 4 | 0 | 4 | 0 | 4 | 0 | |
| Caecum | 4 | 0 | 4 | 0 | 4 | 0 | 4 | 0 | |
| Stomach | 4 | 0 | 4 | 0 | 4 | 0 | 4 | 0 | |
| Bladder | 3 | 0 | 4 | 0 | 4 | 0 | 4 | 0 | |
| Skin | 4 | 0 | 4 | 0 | 4 | 0 | 4 | 0 | |
| Muscle | 4 | 0 | 4 | 0 | 4 | 0 | 4 | 0 | |
| Eye | 4 | 0 | 4 | 0 | 3 | 1 | 4 | 0 | |
| Brain | 3 | 1 | 2 | 2 | 4 | 0 | 3 | 1 | Ventricular dilatation more frequent in Dp1Tyb mice but considered within normal variation in background mouse strain. |
| Spinal cord | 4 | 0 | 4 | 0 | 4 | 0 | 4 | 0 | |
| Pituitary | 4 | 0 | 4 | 0 | 3 | 1 | 3 | 1 | |
| Sciatic nerve | 4 | 0 | 4 | 0 | 4 | 0 | 4 | 0 | |
| Femur | 4 | 0 | 4 | 0 | 4 | 0 | 4 | 0 | |
| Sternum | 4 | 0 | 4 | 0 | 4 | 0 | 3 | 0 | |
| Nasal cavity | 3 | 1 | 3 | 1 | 4 | 0 | 4 | 0 | |
| Harderian gland | 0 | 0 | 0 | 0 | 4 | 0 | 4 | 0 | |
| Ear | 0 | 0 | 0 | 2 | 4 | 0 | 0 | 4 | Significant lesion. Otitis media in Dp1Tyb mice. |
| Uterus | 4 | 0 | 4 | 0 | | | | | |
| Ovary | 4 | 0 | 4 | 0 | | | | | |
| Testes | | | | | 1 | 3 | 1 | 3 | Tubular atrophy in both genotypes but considered within normal variation in background mouse strain. |
| Seminal vesicles | | | | | 3 | 1 | 4 | 0 | |
| Epididymides | | | | | 4 | 0 | 4 | 0 | |
| Prostate | | | | | 4 | 0 | 4 | 0 | |

Disease Models & Mechanisms • Supplementary information

**Table S5. No significant eye abnormalities in Dp1Tyb mice.** Table shows numbers of Dp1Tyb and WT mice that were assessed as being normal or abnormal for each of the features or pathologies listed following eye examination at 15 weeks of age (cohort 1). There were no significant differences between Dp1Tyb and WT mice.

| | WT | | Dp1Tyb | |
|---|---|---|---|---|
| | **Normal** | **Abnormal** | **Normal** | **Abnormal** |
| **eye** | 19 | 1 | 18 | 0 |
| **bulging eye** | 19 | 0 | 18 | 0 |
| **eye haemorrhage** | 19 | 0 | 18 | 0 |
| **eyelid morphology** | 19 | 0 | 18 | 0 |
| **eyelid closure** | 19 | 0 | 18 | 0 |
| **narrow eye opening** | 19 | 0 | 18 | 0 |
| **cornea** | 19 | 0 | 18 | 0 |
| **corneal opacity** | 18 | 2 | 17 | 1 |
| **corneal vascularization** | 19 | 0 | 18 | 0 |
| **iris/pupil** | 18 | 0 | 18 | 0 |
| **pupil position** | 18 | 0 | 18 | 0 |
| **pupil shape** | 18 | 0 | 18 | 0 |
| **pupil dilation** | 18 | 0 | 18 | 0 |
| **pupil light response** | 18 | 0 | 18 | 0 |
| **iris pigmentation** | 18 | 0 | 18 | 0 |
| **lens** | 18 | 0 | 18 | 0 |
| **lens opacity** | 18 | 1 | 16 | 2 |
| **fusion between cornea & lens** | 18 | 0 | 18 | 0 |
| **synechia** | 18 | 0 | 18 | 0 |
| **retina** | 18 | 0 | 18 | 0 |
| **retinal pigmentation** | 18 | 0 | 18 | 0 |
| **retinal structure** | 18 | 0 | 18 | 0 |
| **optic disc** | 18 | 0 | 18 | 0 |
| **retinal blood vessels** | 18 | 0 | 17 | 1 |
| **retinal blood vessels structure** | 18 | 0 | 18 | 0 |
| **retinal blood vessels pattern** | 18 | 0 | 16 | 2 |
| **persistence of hyaloid vascular system** | 15 | 3 | 18 | 0 |

**Table S6. Comparison of phenotypes between human DS and Dp1Tyb and Dp1Yey mouse models of DS.** Table shows phenotypes seen in human DS and results of related phenotypic analysis in Dp1Tyb and Dp1Yey mice. Orange boxes indicate where the results of the mouse phenotyping are different to human studies, blue boxes for differences between Dp1Tyb and Dp1Yey mice. ND, not determined.

| Human DS | | Dp1Tyb mice | | Dp1Yey mice | |
|---|---|---|---|---|---|
| **Phenotype** | **Reference** | **Phenotype** | **Reference** | **Phenotype** | **Reference** |
| Learning and memory deficits | (Grieco et al., 2015; Lott and Dierssen, 2010) | Learning and memory deficits, and electrophysiological alterations | This study and (Chang et al., 2020) | Learning and memory deficits, and electrophysiological alterations | (Aziz et al., 2018; Goodliffe et al., 2016; Jiang et al., 2015; Nguyen et al., 2018; Pinto et al., 2020; Raveau et al., 2018; Souchet et al., 2019; Yu et al., 2010a; Zhang et al., 2014) |
| Locomotor deficits | (Cardoso et al., 2015; Malak et al., 2015) | Locomotor deficits | This study | Locomotor deficits | (Aziz et al., 2018; Goodliffe et al., 2016; Watson-Scales et al., 2018) |
| Impaired sympathetic innervation | (Fernhall and Otterstetter, 2003; Iellamo et al., 2005) | ND | | Impaired sympathetic innervation | (Patel et al., 2015) |
| Disrupted sleep | (Grieco et al., 2015) | Disrupted sleep | This study | Disrupted sleep | (Levenga et al., 2018) |
| Alzheimer's disease | (Wiseman et al., 2015) | No amyloid deposition | This study | ND | |
| Congenital heart defects | (Vis et al., 2009) | Congenital heart defects | (Lana-Elola et al., 2016) | Congenital heart defects | (Li et al., 2007; Liu et al., 2011) |
| Craniofacial alterations | (Vicente et al., 2020) | Craniofacial alterations | This study and (Toussaint et al., 2021) | Craniofacial alterations | (Starbuck et al., 2014; Takahashi et al., 2020) |
| Decreased stature | (Korenberg et al., 1994; LaCombe and Roper, 2020) | Reduced length of body, tibia and femur | This study and (Thomas et al., 2020) | ND | |
| Decreased bone mineral density | (Baptista et al., 2005; Costa et al., 2018; LaCombe and Roper, 2020) | Decreased bone mineral density | This study and (Thomas et al., 2020) | ND | |
| Increased body fat | (Gutierrez-Hervas et al., 2020) | No change in lean mass or fat mass | This study | No change in lean mass or fat mass | (Menzies et al., 2021) |
| Increased rates of type 1 and type 2 diabetes | (Alexander et al., 2016; Johnson et al., 2019) | Decreased blood glucose in fasting mice | This study | Elevated blood glucose in fasting mice | (Peiris et al., 2016) |
| | | Pre-diabetic state | This study | ND | |
| Transient abnormal myelopoiesis | (Bhatnagar et al., 2016; Garnett et al., 2020) | Increased megakaryocyte-erythrocyte progenitors | This study | Increased megakaryocyte-erythrocyte progenitors | (Liu et al., 2018) |
| | | Macrocytic anaemia, splenomegaly | This study | ND | |
| Increased type I interferon response | (Sullivan et al., 2016) | ND | | Increased type I interferon response | (Sullivan et al., 2016) |
| Hearing deficits | (Kreicher et al., 2018) | Hearing deficits | This study | Hearing deficits | (Bhutta et al., 2013) |
| Altered eye structures (eyelid, iris, cornea, lens, retina) | (Krinsky-McHale et al., 2014) | No changes found | This study | ND | |
| Increased retinal thickness | (Laguna et al., 2013) | ND | | Increased retinal thickness | (Victorino et al., 2020) |
| Reduced $VO_2$, $VCO_2$, breathing rate, RER | (Allison et al., 1995; Boonman et al., 2019; Luke et al., 1994) | Higher $VO_2$, $VCO_2$, breathing rate, and RER | This study | Higher $VO_2$, $VCO_2$ | (Menzies et al., 2021) |
| Muscle hypotonia | | Normal grip strength | This study | ND | |
| Placental abnormalities | (Adams et al., 2020) | ND | | Placental abnormalities | (Adams et al., 2021) |

**REFERENCES**

**Adams, A. D., Guedj, F. and Bianchi, D. W.** (2020). Placental development and function in trisomy 21 and mouse models of Down syndrome: Clues for studying mechanisms underlying atypical development. *Placenta* **89**, 58-66.

**Adams, A. D., Hoffmann, V., Koehly, L., Guedj, F. and Bianchi, D. W.** (2021). Novel insights from fetal and placental phenotyping in 3 mouse models of Down syndrome. *Am J Obstet Gynecol*.

**Alexander, M., Petri, H., Ding, Y., Wandel, C., Khwaja, O. and Foskett, N.** (2016). Morbidity and medication in a large population of individuals with Down syndrome compared to the general population. *Dev Med Child Neurol* **58**, 246-254.

**Allison, D. B., Gomez, J. E., Heshka, S., Babbitt, R. L., Geliebter, A., Kreibich, K. and Heymsfield, S. B.** (1995). Decreased resting metabolic rate among persons with Down Syndrome. *Int J Obes Relat Metab Disord* **19**, 858-861.

**Aziz, N. M., Guedj, F., Pennings, J. L. A., Olmos-Serrano, J. L., Siegel, A., Haydar, T. F. and Bianchi, D. W.** (2018). Lifespan analysis of brain development, gene expression and behavioral phenotypes in the Ts1Cje, Ts65Dn and Dp(16)1/Yey mouse models of Down syndrome. *Dis Model Mech* **11**.

**Baptista, F., Varela, A. and Sardinha, L. B.** (2005). Bone mineral mass in males and females with and without Down syndrome. *Osteoporos Int* **16**, 380-388.

**Bhatnagar, N., Nizery, L., Tunstall, O., Vyas, P. and Roberts, I.** (2016). Transient Abnormal Myelopoiesis and AML in Down Syndrome: an Update. *Curr Hematol Malig Rep* **11**, 333-341.

**Bhutta, M. F., Cheeseman, M. T., Herault, Y., Yu, Y. E. and Brown, S. D.** (2013). Surveying the Down syndrome mouse model resource identifies critical regions responsible for chronic otitis media. *Mamm Genome* **24**, 439-445.

**Boonman, A. J. N., Schroeder, E. C., Hopman, M. T. E., Fernhall, B. O. and Hilgenkamp, T. I. M.** (2019). Cardiopulmonary Profile of Individuals with Intellectual Disability. *Med Sci Sports Exerc* **51**, 1802-1808.

**Cardoso, A. C., Campos, A. C., Santos, M. M., Santos, D. C. and Rocha, N. A.** (2015). Motor performance of children with Down syndrome and typical development at 2 to 4 and 26 months. *Pediatr Phys Ther* **27**, 135-141.

**Chang, P., Bush, D., Schorge, S., Good, M., Canonica, T., Shing, N., Noy, S., Wiseman, F. K., Burgess, N., Tybulewicz, V. L. J., et al.** (2020). Altered Hippocampal-Prefrontal Neural Dynamics in Mouse Models of Down Syndrome. *Cell Rep* **30**, 1152-1163.

**Costa, R., Gullon, A., De Miguel, R., de Asua, D. R., Bautista, A., Garcia, C., Suarez, C., Castaneda, S. and Moldenhauer, F.** (2018). Bone Mineral Density Distribution Curves in Spanish Adults With Down Syndrome. *J Clin Densitom* **21**, 493-500.

**Fernhall, B. and Otterstetter, M.** (2003). Attenuated responses to sympathoexcitation in individuals with Down syndrome. *J Appl Physiol* **94**, 2158-2165.

**Garnett, C., Cruz Hernandez, D. and Vyas, P.** (2020). GATA1 and cooperating mutations in myeloid leukaemia of Down syndrome. *IUBMB Life* **72**, 119-130.

**Goodliffe, J. W., Olmos-Serrano, J. L., Aziz, N. M., Pennings, J. L., Guedj, F., Bianchi, D. W. and Haydar, T. F.** (2016). Absence of Prenatal Forebrain Defects in the Dp(16)1Yey/+ Mouse Model of Down Syndrome. *J Neurosci* **36**, 2926-2944.

**Grieco, J., Pulsifer, M., Seligsohn, K., Skotko, B. and Schwartz, A.** (2015). Down syndrome: Cognitive and behavioral functioning across the lifespan. *Am J Med Genet C Semin Med Genet* **169**, 135-149.

**Gutierrez-Hervas, A., Gomez-Martinez, S., Izquierdo-Gomez, R., Veiga, O. L., Perez-Bey, A., Castro-Pinero, J. and Marcos, A.** (2020). Inflammation and fatness in adolescents with and without Down syndrome: UP & DOWN study. *J Intellect Disabil Res* **64**, 170-179.

**Iellamo, F., Galante, A., Legramante, J. M., Lippi, M. E., Condoluci, C., Albertini, G. and Volterrani, M.** (2005). Altered autonomic cardiac regulation in individuals with Down syndrome. *Am J Physiol Heart Circ Physiol* **289**, H2387-2391.

**Jiang, X., Liu, C., Yu, T., Zhang, L., Meng, K., Xing, Z., Belichenko, P. V., Kleschevnikov, A. M., Pao, A., Peresie, J., et al.** (2015). Genetic dissection of the Down syndrome critical region. *Hum Mol Genet* **24**, 6540-6551.

**Johnson, M. B., De Franco, E., Greeley, S. A. W., Letourneau, L. R., Gillespie, K. M., International, D. S. P. C., Wakeling, M. N., Ellard, S., Flanagan, S. E., Patel, K. A., et al.** (2019). Trisomy 21 Is a Cause of Permanent Neonatal Diabetes That Is Autoimmune but Not HLA Associated. *Diabetes* **68**, 1528-1535.

**Korenberg, J. R., Chen, X. N., Schipper, R., Sun, Z., Gonsky, R., Gerwehr, S., Carpenter, N., Daumer, C., Dignan, P., Disteche, C., et al.** (1994). Down syndrome phenotypes: the consequences of chromosomal imbalance. *Proc Natl Acad Sci U S A* **91**, 4997-5001.

**Kreicher, K. L., Weir, F. W., Nguyen, S. A. and Meyer, T. A.** (2018). Characteristics and Progression of Hearing Loss in Children with Down Syndrome. *J Pediatr* **193**, 27-33 e22.

**Krinsky-McHale, S. J., Silverman, W., Gordon, J., Devenny, D. A., Oley, N. and Abramov, I.** (2014). Vision deficits in adults with Down syndrome. *J Appl Res Intellect Disabil* **27**, 247-263.

**LaCombe, J. M. and Roper, R. J.** (2020). Skeletal dynamics of Down syndrome: A developing perspective. *Bone* **133**, 115215.

**Laguna, A., Barallobre, M. J., Marchena, M. A., Mateus, C., Ramirez, E., Martinez-Cue, C., Delabar, J. M., Castelo-Branco, M., de la Villa, P. and Arbones, M. L.** (2013). Triplication of DYRK1A causes retinal structural and functional alterations in Down syndrome. *Hum Mol Genet* **22**, 2775-2784.

**Lana-Elola, E., Watson-Scales, S., Slender, A., Gibbins, D., Martineau, A., Douglas, C., Mohun, T., Fisher, E. M. and Tybulewicz, V.** (2016). Genetic dissection of Down

Disease Models & Mechanisms • Supplementary information

syndrome-associated congenital heart defects using a new mouse mapping panel. *Elife* **5**, 10.7554/eLife.11614.

**Levenga, J., Peterson, D. J., Cain, P. and Hoeffer, C. A.** (2018). Sleep Behavior and EEG Oscillations in Aged Dp(16)1Yey/+ Mice: A Down Syndrome Model. *Neuroscience* **376**, 117-126.

**Li, Z., Yu, T., Morishima, M., Pao, A., LaDuca, J., Conroy, J., Nowak, N., Matsui, S., Shiraishi, I. and Yu, Y. E.** (2007). Duplication of the entire 22.9-Mb human chromosome 21 syntenic region on mouse chromosome 16 causes cardiovascular and gastrointestinal abnormalities. *Hum Mol Genet* **16**, 1359-1366.

**Liu, C., Morishima, M., Yu, T., Matsui, S. I., Zhang, L., Fu, D., Pao, A., Costa, A. C., Gardiner, K. J., Cowell, J. K., et al.** (2011). Genetic analysis of Down syndrome-associated heart defects in mice. *Hum Genet*.

**Liu, C., Yu, T., Xing, Z., Jiang, X., Li, Y., Pao, A., Mu, J., Wallace, P. K., Stoica, G., Bakin, A. V., et al.** (2018). Triplications of human chromosome 21 orthologous regions in mice result in expansion of megakaryocyte-erythroid progenitors and reduction of granulocyte-macrophage progenitors. *Oncotarget* **9**, 4773-4786.

**Lott, I. T. and Dierssen, M.** (2010). Cognitive deficits and associated neurological complications in individuals with Down's syndrome. *Lancet Neurol* **9**, 623-633.

**Luke, A., Roizen, N. J., Sutton, M. and Schoeller, D. A.** (1994). Energy expenditure in children with Down syndrome: correcting metabolic rate for movement. *J Pediatr* **125**, 829-838.

**Malak, R., Kostiukow, A., Krawczyk-Wasielewska, A., Mojs, E. and Samborski, W.** (2015). Delays in Motor Development in Children with Down Syndrome. *Med Sci Monit* **21**, 1904-1910.

**Menzies, C., Naz, S., Patten, D., Alquier, T., Bennett, B. M. and Lacoste, B.** (2021). Distinct Basal Metabolism in Three Mouse Models of Neurodevelopmental Disorders. *eNeuro* **8**.

Disease Models & Mechanisms • Supplementary information

**Nguyen, T. L., Duchon, A., Manousopoulou, A., Loaec, N., Villiers, B., Pani, G., Karatas, M., Mechling, A. E., Harsan, L. A., Limanton, E., et al.** (2018). Correction of cognitive deficits in mouse models of Down syndrome by a pharmacological inhibitor of DYRK1A. *Dis Model Mech* **11**.

**Patel, A., Yamashita, N., Ascano, M., Bodmer, D., Boehm, E., Bodkin-Clarke, C., Ryu, Y. K. and Kuruvilla, R.** (2015). RCAN1 links impaired neurotrophin trafficking to aberrant development of the sympathetic nervous system in Down syndrome. *Nat Commun* **6**, 10119.

**Peiris, H., Duffield, M. D., Fadista, J., Jessup, C. F., Kashmir, V., Genders, A. J., McGee, S. L., Martin, A. M., Saiedi, M., Morton, N., et al.** (2016). A Syntenic Cross Species Aneuploidy Genetic Screen Links RCAN1 Expression to beta-Cell Mitochondrial Dysfunction in Type 2 Diabetes. *PLoS Genet* **12**, e1006033.

**Pinto, B., Morelli, G., Rastogi, M., Savardi, A., Fumagalli, A., Petretto, A., Bartolucci, M., Varea, E., Catelani, T., Contestabile, A., et al.** (2020). Rescuing Over-activated Microglia Restores Cognitive Performance in Juvenile Animals of the Dp(16) Mouse Model of Down Syndrome. *Neuron*.

**Raveau, M., Polygalov, D., Boehringer, R., Amano, K., Yamakawa, K. and McHugh, T. J.** (2018). Alterations of in vivo CA1 network activity in Dp(16)1Yey Down syndrome model mice. *Elife* **7**, 10.7554/eLife.31543.

**Souchet, B., Duchon, A., Gu, Y., Dairou, J., Chevalier, C., Daubigney, F., Nalesso, V., Creau, N., Yu, Y., Janel, N., et al.** (2019). Prenatal treatment with EGCG enriched green tea extract rescues GAD67 related developmental and cognitive defects in Down syndrome mouse models. *Sci Rep* **9**, 3914.

**Starbuck, J. M., Dutka, T., Ratliff, T. S., Reeves, R. H. and Richtsmeier, J. T.** (2014). Overlapping trisomies for human chromosome 21 orthologs produce similar effects on skull and brain morphology of Dp(16)1Yey and Ts65Dn mice. *Am J Med Genet A* **164A**, 1981-1990.

Disease Models & Mechanisms • Supplementary information

**Sullivan, K. D., Lewis, H. C., Hill, A. A., Pandey, A., Jackson, L. P., Cabral, J. M., Smith, K. P., Liggett, L. A., Gomez, E. B., Galbraith, M. D., et al.** (2016). Trisomy 21 consistently activates the interferon response. *Elife* **5**.

**Takahashi, T., Sakai, N., Iwasaki, T., Doyle, T. C., Mobley, W. C. and Nishino, S.** (2020). Detailed evaluation of the upper airway in the Dp(16)1Yey mouse model of Down syndrome. *Sci Rep* **10**, 21323.

**Thomas, J. R., LaCombe, J., Long, R., Lana-Elola, E., Watson-Scales, S., Wallace, J. M., Fisher, E. M. C., Tybulewicz, V. L. J. and Roper, R. J.** (2020). Interaction of sexual dimorphism and gene dosage imbalance in skeletal deficits associated with Down syndrome. *Bone* **136**, 115367.

**Toussaint, N., Redhead, Y., Vidal-Garcia, M., Lo Vercio, L., Liu, W., Fisher, E. M. C., Hallgrímsson, B., Tybulewicz, V. L. J., Schnabel, J. A. and Green, J. B. A.** (2021). A landmark-free morphometrics pipeline for high-resolution phenotyping: application to a mouse model of Down Syndrome. *Development* **148**, dev188631.

**Vicente, A., Bravo-Gonzalez, L. A., Lopez-Romero, A., Munoz, C. S. and Sanchez-Meca, J.** (2020). Craniofacial morphology in down syndrome: a systematic review and meta-analysis. *Sci Rep* **10**, 19895.

**Victorino, D. B., Scott-McKean, J. J., Johnson, M. W. and Costa, A. C. S.** (2020). Quantitative Analysis of Retinal Structure and Function in Two Chromosomally Altered Mouse Models of Down Syndrome. *Invest Ophthalmol Vis Sci* **61**, 25.

**Vis, J. C., Duffels, M. G., Winter, M. M., Weijerman, M. E., Cobben, J. M., Huisman, S. A. and Mulder, B. J.** (2009). Down syndrome: a cardiovascular perspective. *J Intellect Disabil Res* **53**, 419-425.

**Watson-Scales, S., Kalmar, B., Lana-Elola, E., Gibbins, D., La Russa, F., Wiseman, F., Williamson, M., Saccon, R., Slender, A., Olerinyova, A., et al.** (2018). Analysis of motor dysfunction in Down Syndrome reveals motor neuron degeneration. *PLoS Genet* **14**, e1007383.

Disease Models & Mechanisms • Supplementary information

**Wiseman, F. K., Al-Janabi, T., Hardy, J., Karmiloff-Smith, A., Nizetic, D., Tybulewicz, V. L., Fisher, E. M. and Strydom, A.** (2015). A genetic cause of Alzheimer disease: mechanistic insights from Down syndrome. *Nat Rev Neurosci* **16**, 564-574.

**Yu, T., Clapcote, S. J., Liu, C., Li, S., Pao, A., Bechard, A. R., Belichenko, P., Kleschevnikov, A., Asrar, S., Chen, R., et al.** (2010). Effects of individual segmental trisomies of human chromosome 21 syntenic regions on hippocampal long-term potentiation and cognitive behaviors in mice. *Brain Res* **1366**, 162-171.

**Zhang, L., Meng, K., Jiang, X., Liu, C., Pao, A., Belichenko, P. V., Kleschevnikov, A. M., Josselyn, S., Liang, P., Ye, P., et al.** (2014). Human chromosome 21 orthologous region on mouse chromosome 17 is a major determinant of Down syndrome-related developmental cognitive deficits. *Hum Mol Genet* **23**, 578-589.

Disease Models & Mechanisms • Supplementary information
